# True and masked three-coordinate T-shaped platinum(II) intermediates

**DOI:** 10.3762/bjoc.9.153

**Published:** 2013-07-09

**Authors:** Manuel A Ortuño, Salvador Conejero, Agustí Lledós

**Affiliations:** 1 Departament de Química, Universitat Autònoma de Barcelona, 08193 Cerdanyola del Vallès, Spain; 2 Instituto de Investigaciones Químicas (IIQ), Departamento de Química Inorgánica, CSIC and Universidad de Sevilla, Avda. Américo Vespucio 49, 41092 Sevilla, Spain

**Keywords:** C–H bond activation, intermediate, platinum(II), reactive intermediates, three-coordinate, T-shaped

## Abstract

Although four-coordinate square-planar geometries, with a formally 16-electron counting, are absolutely dominant in isolated Pt(II) complexes, three-coordinate, 14-electron Pt(II) complexes are believed to be key intermediates in a number of platinum-mediated organometallic transformations. Although very few authenticated three-coordinate Pt(II) complexes have been characterized, a much larger number of complexes can be described as operationally three-coordinate in a kinetic sense. In these compounds, which we have called masked T-shaped complexes, the fourth position is occupied by a very weak ligand (agostic bond, solvent molecule or counteranion), which can be easily displaced. This review summarizes the structural features of the true and masked T-shaped Pt(II) complexes reported so far and describes synthetic strategies employed for their formation. Moreover, recent experimental and theoretical reports are analyzed, which suggest the involvement of such intermediates in reaction mechanisms, particularly C–H bond-activation processes.

## Review

### Scope of this review

Reaction intermediates are transient species able to undergo transformations along chemical processes. Electron deficient transition-metal complexes with vacant coordination sites are well-suited to play such a role. Coordinatively and electronically unsaturated species have often been invoked as crucial intermediates in reactions involving late transition-metal complexes. Ligand dissociation, forming intermediates with open coordination sites, has been proposed as the initial step in many reactions involving square-planar d^8^ organometallic complexes [1]. Four-coordinate square-planar structures, with a formally 16-electron counting, are absolutely dominant in isolated Pt(II) complexes. However, three-coordinate, 14-electron Pt(II) complexes are believed to be key intermediates in a number of reactions, e.g., β-hydrogen elimination, thermal decomposition of dialkyls, insertion of olefins into M–H bonds, electrophilic attack at Pt–C bonds, and ligand cycloplatination [2]. Likewise, related Pd(II) complexes [3–4] are relevant in cross-coupling reactions and C–H bond-activation processes. The accessibility of three-coordinate Pd(II) species have been recently discussed [5].

Three-coordinate Pt(II) intermediates are the focus of this review. Despite the kinetic perception of the intermediacy of these coordinatively unsaturated species in important organometallic processes, direct proofs of 14-electron Pt(II) complexes have been difficult to find. The strong readiness to alleviate the unsaturation makes them very reactive species but hampers their isolation. Low-coordinate Pt(II) complexes have been generated in gas-phase experiments [6–7] but have remained elusive in solution. With a few exceptions the fourth coordination site is occupied by a weak ligand, e.g., an agostic interaction, a counteranion or a solvent molecule. However, if this additional interaction is weak and labile enough, the three-coordinate species is very accessible and the complex can be considered as “operationally unsaturated” in a kinetic sense [8]. We will name these compounds “masked” three-coordinate complexes to distinguish them from the true low-coordinate complexes. In this review, we will summarize recent advances in true and masked three-coordinate Pt(II) complexes, highlighting both their structural features and their possible participation as reaction intermediates. Computational studies have become an invaluable tool for the investigation of short-lived elusive intermediates and will be quoted throughout the article. The review is organized as follows. First, a general picture of the electronic and geometrical structure of three-coordinate Pt(II) complexes will be presented. Then, the structural features of the main families of these compounds will be summarized. The next sections will be devoted to the spectroscopic tools for their detection and the synthetic strategies employed to their formation. Afterwards, the rearrangement processes exhibited by the low-coordinate complexes in solution will be discussed. Finally, participation of three-coordinate Pt(II) intermediates in reactions, mainly C–H bond-activation processes and ligand exchanges, will be analyzed. A thorough review on the bonding and stereochemistry of three-coordinate transition-metal compounds was published several years ago [9].

### Electronic and geometrical structure

**Figure 1:**
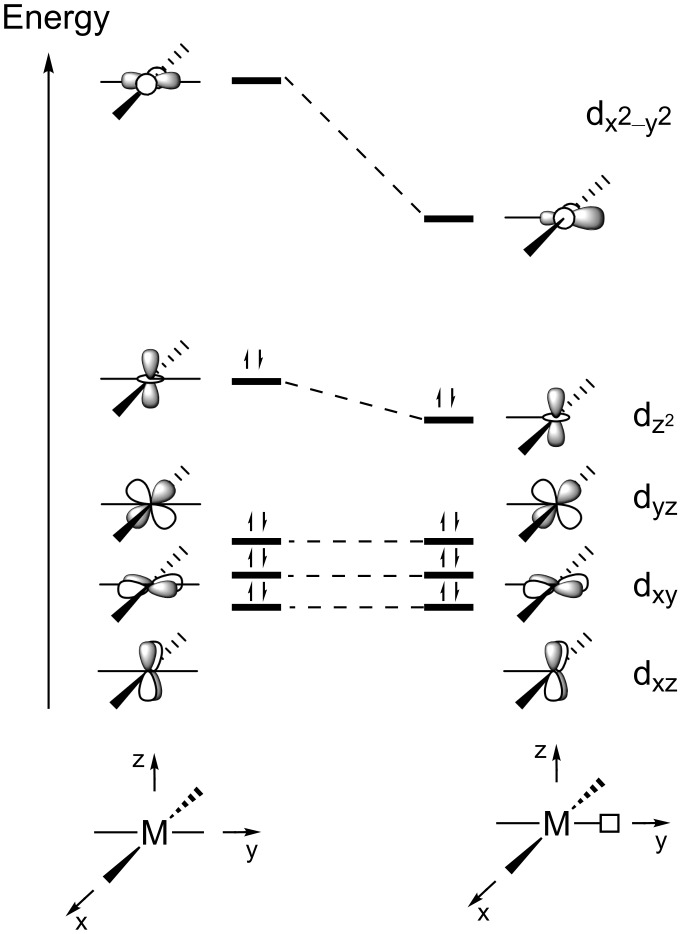
Qualitative orbital diagram for a d^8^ metal in ML_4_ square-planar and ML_3_ T-shaped complexes.

As we will discuss later on, three-coordinate, 14-electron Pt(II) d^8^ complexes display a T-shaped structure, i.e., a structure with two ligands mutually *trans* and the third ligand *trans* to the vacant position. A qualitative molecular orbital scheme of the d block of a T-shaped d^8^ metal complex can be easily derived from that of a square-planar complex by removing one of the ligands (Figure 1) [10–11]. The three nonbonding orbitals in square-planar complexes (d_xz_, d_xy_ and d_yz_) are not affected by the removal of a ligand. The d_z_^2^ orbital is slightly stabilized due to the disappearance of a small antibonding interaction. However, the most striking difference is observed in the lowest unoccupied d_x_^2^_−y_^2^ orbital; its energy strongly decreases by removing an antibonding interaction with one ligand and its shape changes by mixing with the p_y_ orbital. This hybridization takes place so that the orbital is directed away from the three ligands toward the empty coordination site, making this position suitable for the approach of an incoming ligand. This simple picture has been corroborated by DFT calculations on T-shaped [Pt(alkyl)(PMe_3_)_2_]^+^ complexes [2].

The splitting of the d-block orbitals favors a singlet ground state for T-shaped d^8^ complexes and these species usually exhibit a low-spin reactivity. Recent DFT calculations on the 14-electron [Pt(^F^PNP)]^+^ complex (^F^PNP = (4-F-2-(iPr_2_P)C_6_H_3_)_2_N) predict a relatively small (10–12 kcal mol^−1^) separation between the singlet and the triplet states of this intermediate [12]. It is worth pointing out that a d^8^ *C*_2_*_v_* ML_3_ fragment is isolobal with CH_2_, although the ordering of the two valence orbitals a_1_ and b_2_ differs. For a singlet 14-electron T-shaped ML_3_, the two electrons fill the b_2_ level (d_yz_), while for a singlet CH_2_ fragment they occupy the a_1_ orbital (empty d_x_^2^_−y_^2^ in ML_3_, Figure 1) [13].

**Figure 2:**
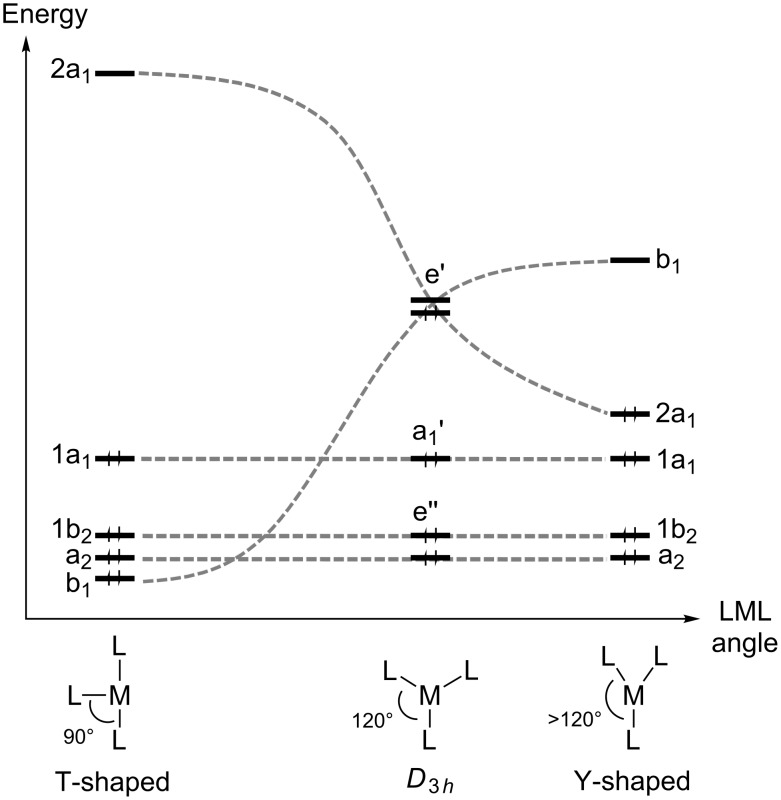
Walsh diagram for the d-block of a d^8^ ML_3_ complex upon bending of one L–M–L angle.

Steric reasons should favor trigonal-planar *D*_3_*_h_*-like structures for three-coordinate Pt(II) complexes instead of the sterically unfavorable T-shaped structure. The preference of these Pt(II) compounds (with a low spin d^8^ configuration) for T-shaped structures is due to electronic effects and can be understood from the Walsh diagram shown in Figure 2. The Walsh diagram describes the variation of the L–M–L angle from a T-shaped structure through *D*_3_*_h_* to a Y-shaped structure. A d^8^, 14-electron ML_3_ complex would have the lowest four levels filled in Figure 2. In a *D*_3_*_h_* geometry, the degeneracy of e’ orbitals will promote a Jahn–Teller distortion towards T or Y geometries [10,14].

DFT calculations on asymmetric *cis-*[Pt(alkyl)(PMe_3_)_2_]^+^ show that, in spite of the reduced symmetry, the bending energy profile maintains the same basic features: the T-shaped configurations of the 14-electron species are energy minima, and their *cis*-like to *trans*-like interconversion occurs via transition states with Y-shaped configurations [2].

**Figure 3:**
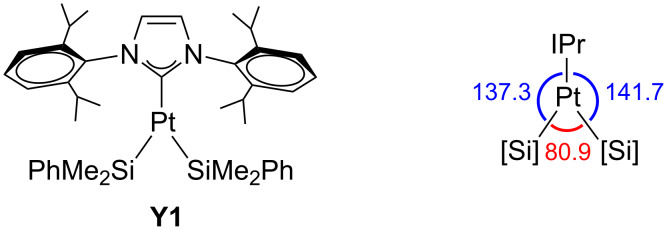
Neutral Y-shaped Pt complex **Y1** [15]. Angles are given in degrees.

The X-ray structure of the recently reported three-coordinate platinum complex [Pt(SiMe_2_Ph)_2_(IPr)] (IPr = 1,3-bis(2,6-diisopropylphenyl)imidazol-2-ylidene) shows a unique Y-shaped geometry in which the Si–Pt–Si angle is very acute (80.9°) and far from the ideal values for both trigonal-planar and T-shaped structures (**Y1**, Figure 3) [15]. Computations on non-sterically demanding models [Pt(R)_2_(Im)] (R = SiMe_3_, Me; Im = imidazol-2-ylidene) appealed to the *trans* influence of both NHC and silyl ligands to explain the structure. However, a recent DFT investigation concluded that **Y1** is better described as a Pt(0) σ-disilane complex [16] than as a Pt(II) disilyl species. Thorough geometrical and electronic analyses support a Pt(0)···disilane coordination via donation and back-donation interactions. This suggestion also explains the experimentally observed ^195^Pt NMR chemical shift, which is closer to the Pt(0) rather than the Pt(II) species.

The nonequivalence of axial and equatorial positions in ML_3_ complexes raises the question of which positions are preferred by the ligands in such compounds. Although in most of the experimentally characterized systems the steric demands dictate the disposition of the ligands, electronic effects are also at work, and they were analyzed for simple model systems with theoretical methods. An early study based on perturbation theory concluded that in T-shaped ML_3_ d^8^ complexes both axial and equatorial bonds should have similar strength and that the most electronegative atoms will substitute axially [17]. DFT calculations on [PtXY(PH_3_)] (X, Y = Cl, CH_3_, SiH_3_, Si(OH)_3_) demonstrated the importance of the *trans* influence in governing the stability of T-shaped isomers. In the most stable isomer, the ligand with the smaller *trans* influence is located *trans* to the PH_3_ ligand [18].

### Structurally characterized compounds

The T-shaped structure is easily recognized by the absence of one ligand in a square-planar disposition. Nevertheless, the number of well-characterized three-coordinate Pt(II) complexes is very low. To achieve a true T-shaped structure, the vacancy at the metal center must be blocked to avoid intra- and intermolecular interactions, such as agostic bonds and counteranion or solvent coordination. These interactions mask the T-shaped structure, but due to their potential labile nature, the three-coordinate species are still accessible.

**Figure 4:**
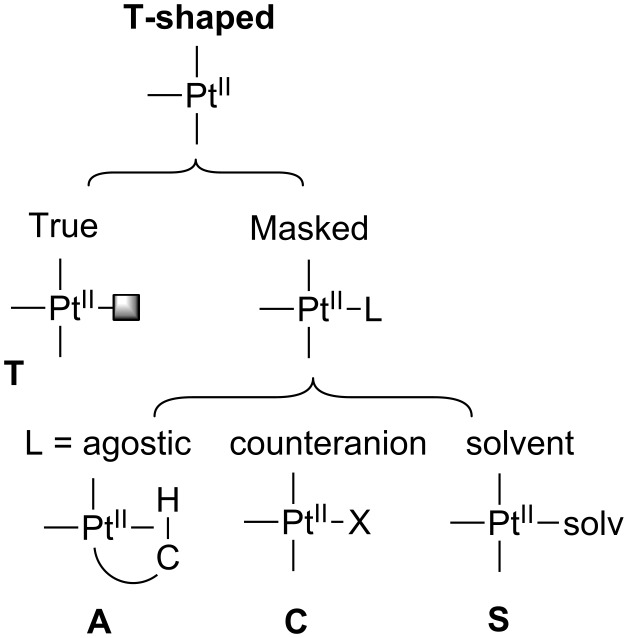
General classification of T-shaped Pt(II) structures according to the fourth coordination site.

In this section we will describe the main families of T-shaped Pt(II) complexes, which are structurally characterized (Figure 4). First, true T-shaped structures with no stabilization at the vacant site are compiled. Then, complexes involving agostic, counteranion and solvent interactions will be summarized. From now on, true T-shaped complexes are labeled as **T** complexes, while agostic, counteranion and solvent-stabilized complexes are designated as **A**, **C** and **S** complexes, respectively.

### True T-shaped

The isolation and characterization of such highly electrophilic species is challenging. Accordingly, the number of well-characterized true T-shaped Pt(II) complexes is scarce. The empty site should be blocked somehow to prevent undesirable contacts, and in most cases, bulky ligands are required to protect the empty coordination site. Strong electron-donor ligands also help stabilizing the formally 14-electron compounds. It is worthy of note that almost all of them are cationic.

**Figure 5:**
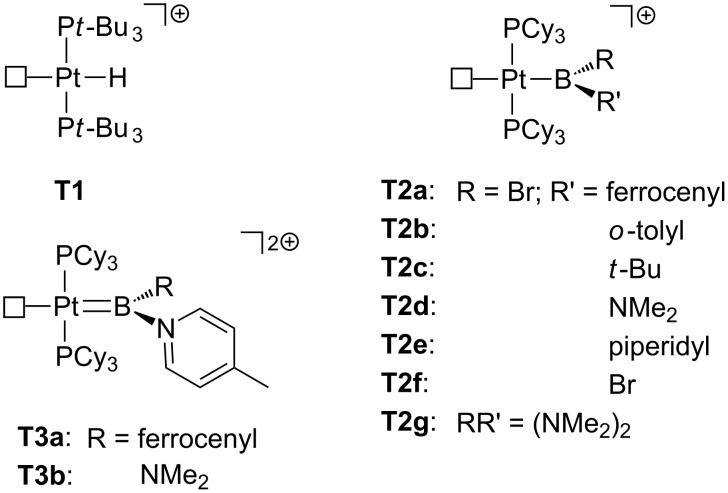
Hydride, boryl and borylene true T-shaped Pt(II) complexes.

The first true T-shaped complexes were prepared by using phosphine ligands (Figure 5). In the 80s, Goel et al. proposed, from nuclear magnetic resonance (NMR) data, a T-shaped structure for the cationic hydride *trans*-[(*t*-Bu_3_P)_2_Pt(H)]^+^ **T1** containing bulky phosphine ligands [19]. However, it was not until 2005 when Braunschweig et al. successfully characterized a true 14-electron T-shaped Pt(II) boryl complex **T2a** by means of X-ray studies [20]. By halide abstraction, the cationic *trans*-[Pt(B(Fc)Br)(PCy_3_)_2_]^+^ **T2a** (Fc = ferrocenyl; Cy = cyclohexyl) could be obtained, in which the boryl ligand is located *trans* to the empty site. No agostic interactions were detected, the shortest Pt–H and Pt–C distances being 2.542 Å and 3.117 Å, respectively. Inspired by this chemical template, the synthesis of Pt–boryl derivatives of the type *trans*-[Pt(BRR’)(PCy_3_)_2_]^+^ has been extended [21]. Even at extreme electronic conditions in dicationic *trans*-[Pt(BR(4-picoline))(PCy_3_)_2_]^2+^ **T3**, the complex remains truly T-shaped, although small Lewis donors such as CO and acetonitrile can coordinate to the open coordination site of **T3** [22]. DFT-based electron localization function (ELF) analyses [20] and geometry optimizations [22] supported the lack of agostic interactions in these complexes. This absence has been attributed to the strong *trans* influence exerted by the ligand in *trans* position with respect to the vacant site [23–24].

**Figure 6:**
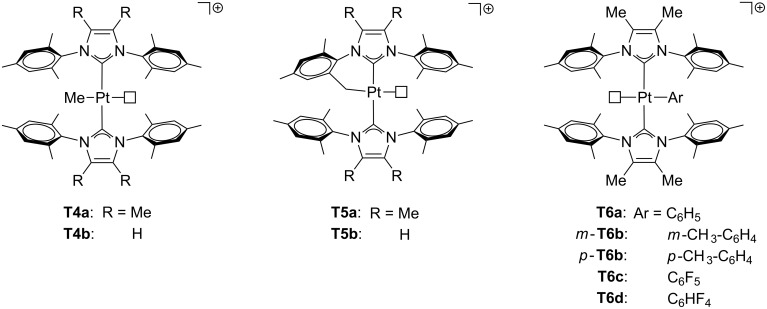
NHC-based true T-shaped Pt(II) complexes.

Moreover, in the past few years the synthesis of 14-electron Pt(II) complexes was extended by using N-heterocyclic carbene (NHC) ligands (Figure 6), which have been proven to be useful stabilizing electron-deficient transition-metal species [25–27]. In this regard, recent studies state that the use of IMes* (4,5-dimethyl-1,3-bis(2,4,6-trimethylphenyl)imidazol-2-ylidene) and IMes (1,3-bis(2,4,6-trimethylphenyl)imidazol-2-ylidene) ligands in [Pt(Me)(NHC)_2_]^+^ **T4** and [Pt(NHC’)(NHC)]^+^ **T5** (NHC = IMes*, IMes; NHC’ = cyclometalated ligand) provides pure T-shaped species with no agostic stabilization [28]. Additionally, the resulting [Pt(Ar)(IMes*)_2_]^+^ **T6** formed after C–H bond activation has also proven to be a three-coordinate species with no agostic interactions according to the X-ray structure of the derivative **T6d**, in which the closest Pt–H contact is located at 3.117 Å. The absence of agostic interactions has been attributed to geometrical constraints, the limited flexibility of the mesityl groups in IMes* and IMes hampering the approach of the CH bond to the metal center. Once again, Lewis acids as acetonitrile can access the empty site, which means that a potential reactant molecule can coordinate to the metal for further reactions.

### Masked T-shaped via agostic interaction

The agostic interaction [29–30] is usually explained as an intramolecular 3-center-2-electron interaction between a metal M and a C–H bond. This type of contact is a recurrent event in unsaturated transition-metal complexes [31] and it can be characterized by structural and spectroscopic techniques [29–30] together with computational tools [32–33]. The agostic interaction shows different behaviors. It can be a transient species prior to the C–H bond breaking or stabilize low electron count situations. In this section we will focus on the latter aspect. Since this intramolecular interaction is difficult to avoid, even in the presence of bulky ligands, it is the most common motif for the stabilization of three-coordinate complexes.

**Figure 7:**
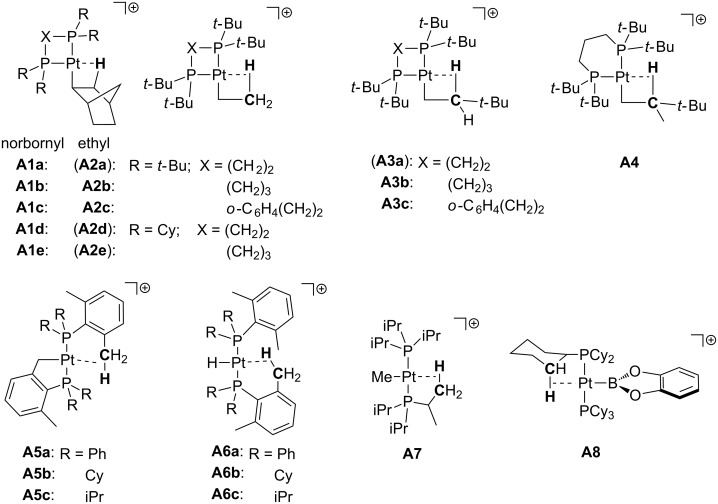
Phosphine-based agostic T-shaped Pt(II) complexes. Compounds in brackets correspond with hydrido–alkene ground states.

Early works on T-shaped Pt(II) species are related to complexes stabilized with phosphine ligands (Figure 7). Pioneering studies on alkyl complexes from Orpen’s and Spencer’s research groups reported the synthesis of cationic [Pt(norbornyl)(P–P)]^+^ complexes **A1a**–**e** (P–P = bidentate phosphine ligand) [34–35]. Further studies involved other alkyl ligands such as ethyl (**A2b**–**c**), 3,3-dimethylbutyl (**A3b**–**c**) and 2,3,3-trimethylbutyl (**A4**) [36–38]. NMR spectroscopic data and X-ray structures of **A1a** [34] and **A2b** [36] demonstrate that these compounds display a β-agostic interaction filling the fourth coordination site. This interaction is strong enough to cleave the C–H bond, creating a chemical equilibrium with the corresponding hydrido–alkene derivatives. Indeed, the detected ground state of the compounds in brackets (**A2a**, **A2d**, **A2e** and **A3a** in Figure 7) is the hydrido–alkene isomer instead of the agostic–alkyl one. Later, Baratta et al. succeeded in the isolation of 14-electron Pt(II) complexes bearing bulky phosphine ligands [39]. The usage of *trans*-[Pt(Me)Cl(PR_3_)_2_] as a starting material, followed by halide removal and the release of methane by intramolecular C–H bond activation provided the cationic T-shaped cyclometalated complexes **A5a**–**b**. The X-ray structure of **A5b** shows that the platinum atom exhibits a δ-agostic interaction with one hydrogen atom of the methyl group of the non-cyclometalated phosphine ligand. Subsequent hydrogenation generates the corresponding hydride complexes **A6a**–**b**. On the basis of NMR studies (^1^*J*_Pt,H_ values of ca. 2000 Hz, see next section), a weak interaction *trans* to the hydride ligand, typically an agostic contact, is supposed to exist. Carmona and co-workers also prepared quite similar complexes, **A5c** and **A6c**, where R labels represent isopropyl groups [40]. Weller and co-workers reported the formation of complex *trans*-[Pt(Me)(PiPr_3_)_2_]^+^ **A7** in which a γ-agostic interaction is located in the fourth coordination site [41]. The addition of tetrahydrofuran (THF) rapidly forms the corresponding adduct. Braunschweig et al. obtained the agostic structure **A8** by employing BCat as a ligand (BCat = catecholatoboryl) in three-coordinate Pt(II) complexes [21]. Unlike the previous pure T-shaped boryl complexes **T2** (Figure 5), the weaker *trans* influence of the BCat ligand allows the formation of an agostic contact. Treatment of **A8** with Lewis bases also removes the agostic interaction, generating the adduct complex.

Natural Bond Orbital (NBO) and Atoms In Molecules (AIM) calculations on complexes **A1**, **A2b**, **A5b** and **A7** classified the Pt···H–C contacts as agostic interactions for all the species with the only exception being **A7**, in which a H-bond character can also be ascribed [42].

**Figure 8:**
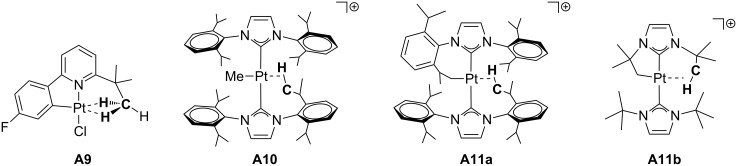
Phenylpyridine and NHC-based agostic T-shaped Pt(II) complexes.

Concerning other types of ligands (Figure 8), the phenylpyridyl complex **A9** reported by Rourke and co-workers exhibits a bifurcated δ-agostic interaction that has been determined by X-ray studies [43]. This is one of the rare neutral T-shaped Pt(II) complexes. Referring to NHC ligands, Rivada-Wheelaghan et al. isolated the three-coordinate methyl [Pt(Me)(IPr)_2_]^+^ (**A10**) and cyclometalated [Pt(NHC’)NHC]^+^ complexes (NHC = IPr **A11a**, I*t*-Bu (1,3-bis(*tert*-butyl)imidazol-2-ylidene) **A11b**; NHC’ = cyclometalated ligand) [44]. In sharp contrast with the analogous **T5** and **T6** (Figure 6) [28], δ- and ζ-agostic interactions at the fourth coordination site were detected by X-ray and NMR studies for complexes containing IPr (**A11a**) and I*t*-Bu (**A11b**), respectively.

Selected Pt···H–C parameters from the X-ray structures of agostic compounds are collected in Table 1. As it can be expected, short Pt–H and Pt–C distances are observed for the β-agostic interactions in **A1a** and **A2b**. In addition, Pt–H–C angles larger than 100° correlate with large Pt–C distances as shown in **A7**, **A8** and **A11a**. Although a remote contact is described in **A11a**, the largest distances correspond with the γ-agostic **A8**. In other words, the strength of the agostic interaction is not only governed by geometrical constraints and the surroundings of the CH group, but also by the *trans* influence of the ligand in *trans* position with respect to the agostic interaction [23–24]. In this case, the boryl ligand in **A8** exhibits a higher *trans* influence than the alkyl group in **A11a** [45–46], and therefore the agostic interaction in the former is weaker.

**Table 1:** Selected geometrical parameters of X-ray characterized agostic T-shaped Pt(II) structures.

Complex	Agostic length	Pt–H distance/Å	Pt–C distance/Å	Pt–H–C angle/degrees	Reference

**A1a**	β	1.895	2.309	91.2	[34]
**A2b**	β	—^a^	2.282	—^a^	[37]
**A5b**	δ	2.057	2.432	102.5	[39]
**A7**	γ	2.244	2.855	124.2	[41]
**A8**	γ	2.322	2.923	118.2	[21]
**A9**	δ	2.151, 2.138^b^	2.472	96.5, 94.6^b^	[42]
**A11a**	ζ	2.017	2.876	145.1	[44]

^a^Not available. ^b^Bifurcated agostic interaction.

### Masked T-shaped via counteranion interaction

**Figure 9:**
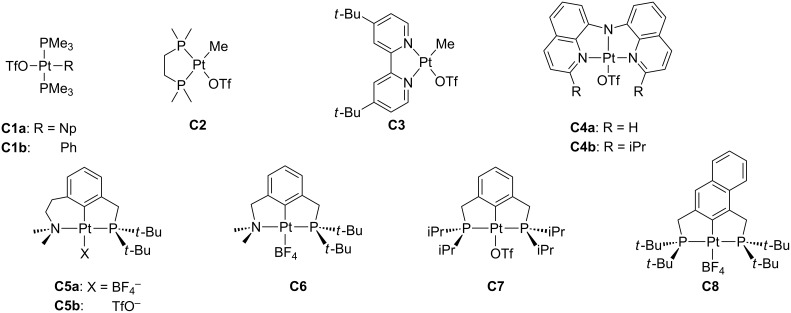
Counteranion coordination in T-shaped Pt(II) complexes.

Most of the T-shaped Pt(II) complexes are cationic. Thus, there is the possibility of stabilizing the unsaturated structure by nonbulky, weakly coordinating counteranions. Triflate (OTf = SO_3_CF_3_) and tetrafluoroborate are the best candidates to play such a role (Figure 9). All the well-characterized compounds bear a coordinated triflate anion. In the 80s, Whitesides et al. described the synthesis of *trans*-[Pt(Np)(OTf)(PMe_3_)_2_] (Np = neopentyl) **C1a**. This compound reacts with benzene forming *trans*-[Pt(Ph)(OTf)(PMe_3_)_2_] **C1b** through a mechanism that involves *trans*-[Pt(Np)(PMe_3_)_2_]^+^ as a reactive intermediate [47]. The labile character of the coordinated triflate has been alleged to justify the significant difference between the ^1^*J*_Pt,P_ found for [Pt(Me)(OTf)(dmpe)] **C2** (dmpe = 1,2-bis(dimethylphosphino)ethane) in CD_2_Cl_2_ (4572 Hz) and in acetone (4305 Hz). In the latter media one solvent molecule is proposed to displace the triflate ligand, forming [Pt(Me)(acetone)(dmpe)][OTf] [48]. The complex [Pt(Me)(OTf)(dbbipy)] **C3** (dbbipy = 4,4'-di-*tert*-butyl-2,2'-bipyridine) was prepared by treatment of [PtCl(Me)(dbbipy)] with AgOTf [49]. Regarding tridentate ligands, the X-ray structure of the triflate complex [Pt(3,3'-iPr_2_-BQA)(OTf)] **C4b** (BQA = bis(8-quinolinyl)amine) shows a coordinated OTf ligand (Pt–O = 2.097 Å) in *trans* position to the amido N-donor of the pincer-like amido ligand. In solution, the labile triflate ligand can be displaced, allowing a reaction with benzene [50]. Milstein and co-workers have reported a series of pincer-type Pt(II) complexes **C5**–**8** containing an XCX ligand core (X = N, P), incorporating the anions OTf^−^ and BF_4_^−^ [51–53]. The structure of **C5b** was determined by X-ray crystallography [51]. The Pt–O bond distance in this compound, in which the triflate anion is coordinated to platinum in *trans* position with respect to the aromatic ring, is considerably longer than in **C4b** (**C4b**: 2.097 Å [50]; **C5b**: 2.249 Å [51]). The weakly coordinating character of triflate accounts for its sensitivity to the *trans* influence.

### Masked T-shaped via solvent coordination

**Figure 10:**
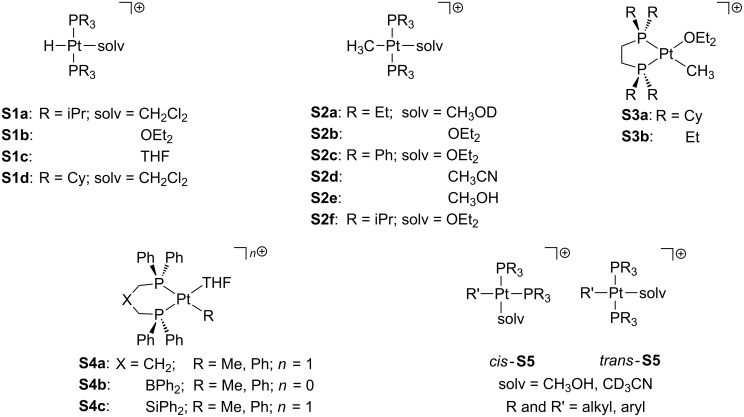
Phosphine-based solvento Pt(II) complexes.

In solution, a solvent molecule can occupy the vacant position of a T-shaped three-coordinate Pt(II) compound via σ-interaction. A number of phosphine-based complexes with different solvent molecules coordinated to the T-shaped frame have been characterized (Figure 10). In this regard, Kubas and co-workers prepared a set of hydride *trans*-[PtH(PiPr_3_)_2_(solv)]^+^ compounds where “solv” stands for η^1^-ClCH_2_Cl (**S1a**), OEt_2_ (**S1b**) and THF (**S1c**) solvent molecules [54]. Interestingly, the closely related species **T1** (Figure 5) does not include any solvent molecule filling the vacancy [19]. Moreover, the structures of **S1a** and **S1c** were fully confirmed by X-ray studies. The similar dichloromethane adduct **S1d** was later detected by NMR spectroscopy [55]. This work was extended to methyl complexes with the formula *trans*-[Pt(Me)(PR_3_)_2_(solv)] (**S2**) [56–57]. Similar structures have been obtained by the use of chelating phosphine ligands in diethylether (**S3**) [58]. In this line, Peters and co-workers developed several bidentate phosphine ligands to yield complexes **S4** in which one THF molecule is coordinated to the metal center [59–60]. Romeo’s group was actively working on solvento Pt(II) complexes **S5**. For instance, they employed an extended series of phosphine ligands to obtain *cis* and *trans-*solvento complexes containing methanol [61]. The formation of acetonitrile-*d*_3_ adducts with triethylphosphine ligands was also reported [2,62].

**Figure 11:**
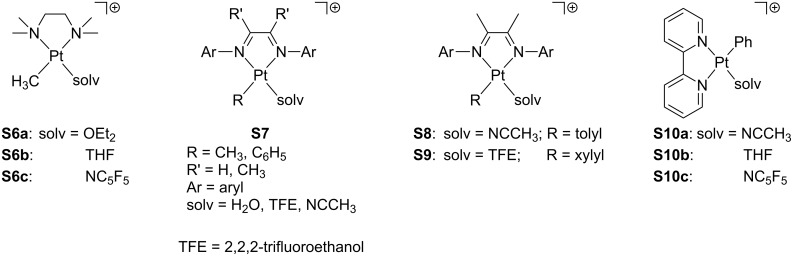
Nitrogen-based solvento Pt(II) complexes.

Several examples involving chelating nitrogen-based ligands have also been reported (Figure 11). Bercaw and co-workers developed cationic solvento Pt(II) complexes **S6** bearing tetramethylethylenediamine (tmeda) [63]. X-ray crystallography of **S6a**·BAr^F^ and **S6c**·BAr^F^ verified the solvent coordination [64]. Diimine analogues, valuable in C–H bond-activation processes, have been extensively reported elsewhere (**S7**) [65–72]. Later, Tilset’s group prepared and characterized a set of solvento tolyl complexes of the type [Pt(Tol)(N–N)(CH_3_CN)] **S8** by means of ^1^H and ^19^F NMR [73]. The formation of *p*-xylene derivates **S9** containing a molecule of the weakly coordinating 2,2,2-trifluoroethanol (TFE) has also been reported [69,74]. Unsaturated bipyridyl compounds **S10** have been isolated in which the presence of solvent molecules has been detected [75–76]. The stabilizing role of the solvent molecule is quite relevant. For instance, the removal of the THF molecule in **S10b** provokes the decomposition of the complex [75]. The facile displacement of the solvent ligand allows the use of these compounds as catalysts in hydrophenylation reactions [76]. Other related complexes have been prepared elsewhere [77–81].

**Figure 12:**
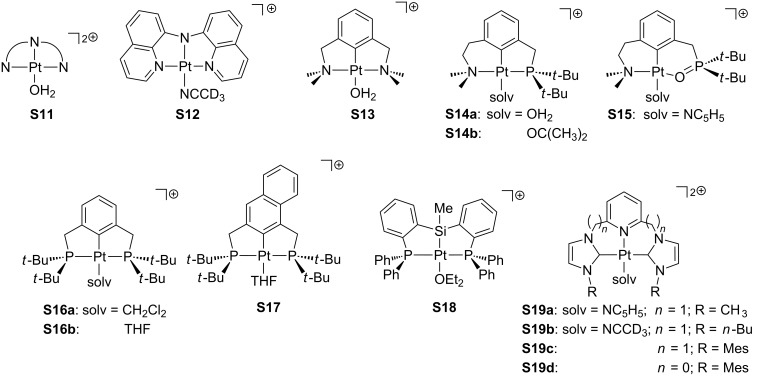
Pincer-based solvento Pt(II) complexes.

Pincer ligands [82–83] naturally define a three-coordinate environment and, in this way, they are well-suited as ligands in T-shaped compounds. Accordingly, a number of T-shaped Pt(II) pincer complexes with an additional solvent molecule as a fourth ligand have been reported (**S11**–**19**, Figure 12). Van Eldik and co-workers studied the aquo complexes **S11** by using several tridentate nitrogen-based ligands (NNN) [84]. Peters and co-workers isolated [Pt(OTf)(NNN)] species, though NMR data were collected in acetonitrile-*d*_3_ solution, in which the solvento adduct **S12** is favored [50]. Extensive work carried out by van Koten and co-workers [85–86] concerned NCN aquo compounds such as **S13**. Unsaturated complexes bearing NCP ligands were characterized by Milstein and co-workers, including water (**S14a**) [51] and acetone (**S14b**) [87] solvent molecules. Isolation of compound **S15** in acetone/pyridine solution provided suitable crystals for X-ray studies, which showed that a pyridine molecule has been added [87]. Other investigations have been devoted to complexes with PCP ligands. For instance, Bullock and co-workers prepared a dihydrogen adduct of the type [PtH_2_(PCP)]^+^ in dichloromethane, but NMR data suggest a mixture of the former compound and the corresponding solvento complex **S16a** [88]. Milstein et al. proposed that the closely related complex **S16b** exists in THF solution, since an interaction with the BF_4_^−^ counteranion is not observed, although the elemental analysis evidences the lack of the THF molecule in the solid state [89]. Contrarily, the naphthyl derivative **S17** was found together with the BF_4_^−^ adduct **C8** (Figure 9) [53]. For silicon-related ligands, Turculet and co-workers successfully crystallized **S18** in a diethylether solution at low temperature, showing one diethylether molecule directly coordinated to the platinum center [90]. A set of dicationic complexes based on carbene groups have been developed by Limbach and co-workers, forming pyridine **S19a** and acetonitrile **S19b**,**c** adducts [91].

**Figure 13:**
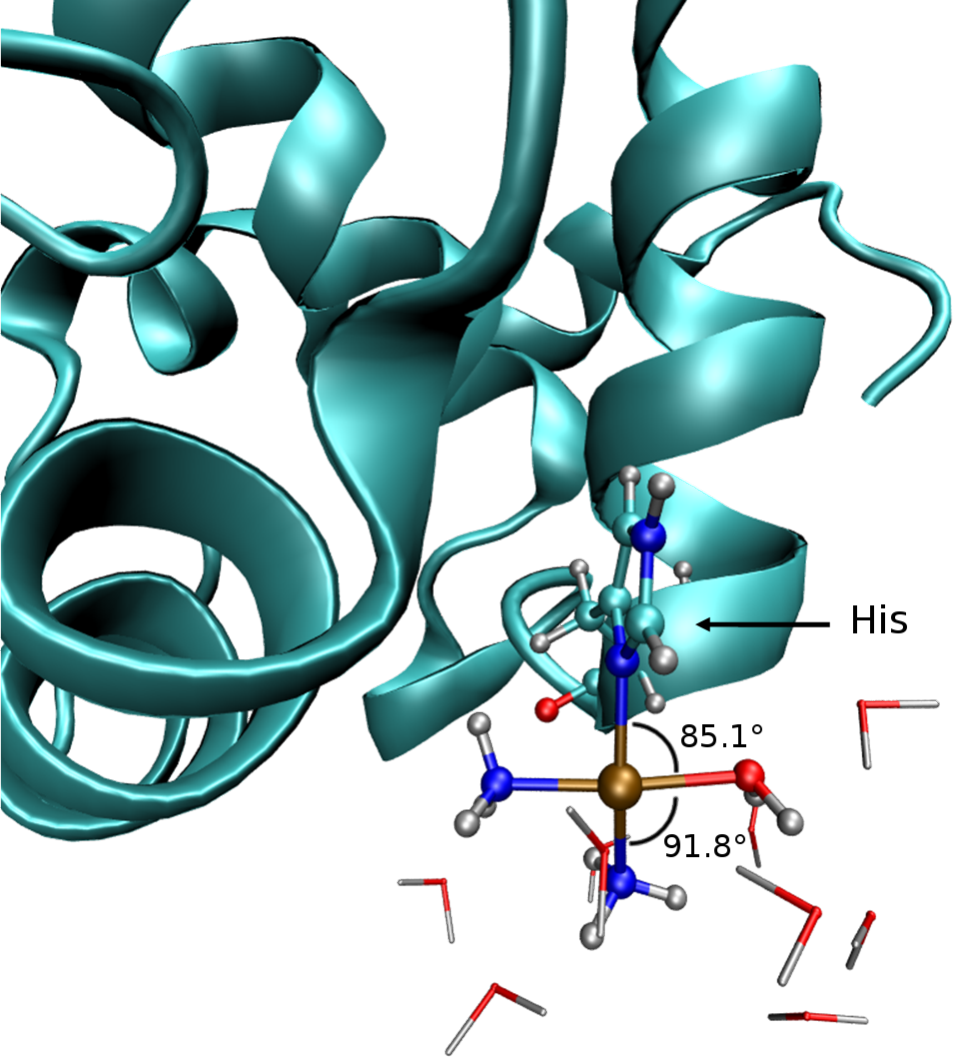
Structure of the QM/MM optimized cisplatin–protein adduct [94].

The relevance of solvent-stabilized T-shaped Pt(II) complexes extends beyond the organometallic field. Their presence in cisplatin–protein adducts has also been proposed concerning the bovine Cu, Zn superoxide dismutase [92] and the hen egg white lysozyme [93]. The corresponding crystallographic structures show the platinum coordinated to a histidine residue of the protein and two ligands (Cl^−^ [92] or NH_3_ [93]). However, in both situations the fourth ligand was not fully detectable, so that the authors suggested the participation of a coordinating water molecule. Indeed, quantum mechanics/molecular mechanics (QM/MM) calculations on the cisplatin–hen egg white lysozyme adduct confirmed the facile inclusion of a solvent water molecule in the first coordination shell of the platinum complex (Figure 13) [94].

### NMR coupling constants to ^195^Pt as sensitive probes for coordinatively unsaturated Pt(II) complexes

**Figure 14:**
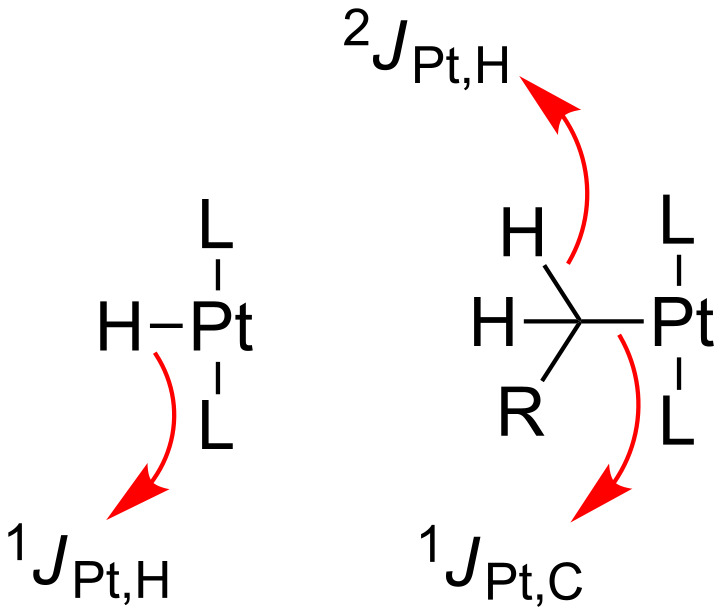
NMR coupling constants used for the characterization of three-coordinate Pt(II) species.

NMR spectroscopy has provided additional evidence for the existence of low electron-count Pt(II) complexes not only through the observation (in some cases) of NMR signals for the C–H bonds involved in agostic interactions, but also by the magnitude of the coupling constant of some of the ligands around the metal center with the NMR active ^195^Pt nuclei (33.7% natural abundance). This is actually not the case with ^31^P NMR, for which the *J*_Pt,P_ values seem to be virtually insensitive to the nature of the complex. As an example, in the boryl derivatives reported by Braunschweig et al. [21] the *J*_Pt,P_ coupling constant is almost the same both in the starting material, [Pt(BR_2_)Br(PCy_3_)_2_], and in the three-coordinate Pt(II) species, [Pt(BR_2_)(PCy_3_)_2_][BAr^F^] **T2**. On the other hand, the coupling constants of the proton and carbon atoms of alkyl and hydride ligands with ^195^Pt are very sensitive to the presence of coordinating ligands *trans* to them (Figure 14).

**Figure 15:**
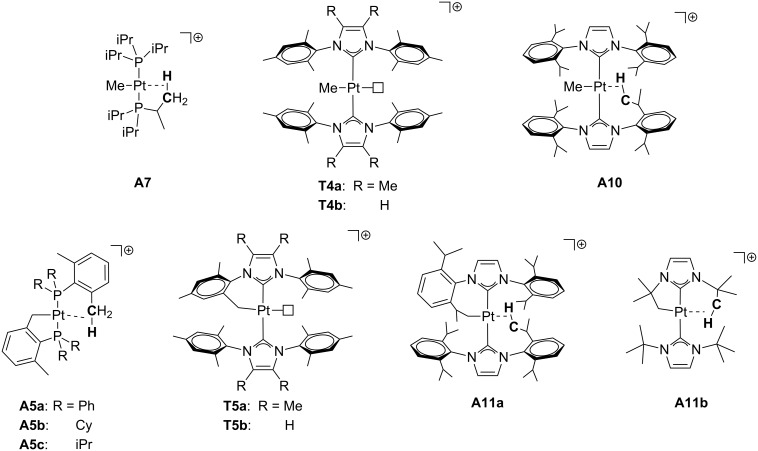
The chemical formula of the complexes discussed in Table 2.

A nice example is illustrated in the hydride, T-shaped Pt(II) complex *trans*-[PtH(P*t*-Bu_3_)_2_][X] **T1** (X = PF_6_, BF_4_, ClO_4_, SO_3_CF_3_) reported by Goel and Srivastava [19]. The magnitude of the ^1^*J*_Pt,H_ coupling constant of the hydride ligand in its precursor *trans*-[PtHCl(P*t*-Bu_3_)_2_] is 1070 Hz, but in the 14-electron derivative *trans*-[PtH(P*t*-Bu_3_)_2_][X] **T1** it increases to ca. 2600 Hz, the largest ^1^*J*_Pt,H_ reported in the literature. A decrease of this coupling constant to 2050–2070 Hz was observed in the related hydride Pt(II) complexes *trans*-[PtH(PR_2_(2,6-Me_2_C_6_H_3_))_2_]^+^ (Figure 7, R = Ph **A6a**, Cy **A6b**, iPr **A6c**), which are stabilized by agostic interactions involving the methyl groups of the aryl fragments. In the same vein, weakly coordinating ligands such as the solvent stabilized Pt(II) derivatives *trans*-[PtH(ClCH_2_Cl)(PR_3_)_2_][BAr^F^] (Figure 10, R = iPr **S1a**, Cy **S1d**) exhibit a smaller *J*_Pt,H_ of 1480 (R = Cy) or 1852 Hz (R = iPr) in agreement with the presence of a ligand *trans* to the hydride. With regard to Pt–H two-bond and Pt–C one-bond coupling constants in Pt–C_alkyl_ complexes (Figure 15 and Table 2), Weller et al. reported that the methyl group in the complex *trans*-[Pt(Me)(PiPr_3_)_2_]^+^ **A7** exhibits a ^2^*J*_Pt,H_ value of 106 Hz. This value is almost identical to those reported for the NHC derivatives *trans*-[Pt(Me)(NHC)_2_]^+^ (NHC = IMes* **T4a**, IMes **T4b**; 110 and 103 Hz, respectively). The ^1^*J*_Pt,C_ is also very large for these methyl derivatives, ranging from 755 to 780 Hz (Table 2). Cyclometalated phosphine [39–40] and NHC [28,44] Pt(II) compounds were also shown to have very large ^2^*J*_Pt,H_ coupling constants (slightly larger than those for the non-cyclometalated versions) spanning from 107 Hz to ca. 135 Hz (average) and ^1^*J*_Pt,C_ values between 805 and 975 Hz.

**Table 2:** NMR coupling constants of selected three-coordinate Pt(II) complexes.

Complex	^2^*J*_Pt,H_/Hz	^1^*J*_Pt,C_/Hz	Reference

**A7**	106	755	[41]
**T4a**	103	—^a^	[28]
**T4b**	110	—^a^	[28]
**A10**	—^a^	780	[44]
**A5a**	109	840	[39]
**A5b**	107	806	[39]
**A5c**	107	—^a^	[40]
**T5a**	132 (av.)	—^a^	[28]
**T5b**	135	—^a^	[28]
**A11a**	113 (av.)	860	[44]
**A11b**	120	975	[44]

^a^Not available.

In those cases where solvent molecules (THF, acetonitrile) have been reported to coordinate to some of the complexes shown in Figure 15, smaller values of both the ^2^*J*_Pt,H_ and the ^1^*J*_Pt,C_ coupling constant are observed. For example, the ^2^*J*_Pt,H_ of the methyl ligand decreases to 98 Hz in the THF adduct of derivative **A7**, whereas the acetonitrile adduct of complex **A11b** (**A11b·**NCMe) shows resonances for the Pt–CH_2_ fragment with ^2^*J*_Pt,H_ and ^1^*J*_Pt,C_ values of 87 and 798 Hz, respectively [28].

### Synthetic routes to stable, solvent-stabilized and transient 14-electron Pt(II) species

#### True and agostic T-shaped Pt(II) complexes

**Scheme 1:**
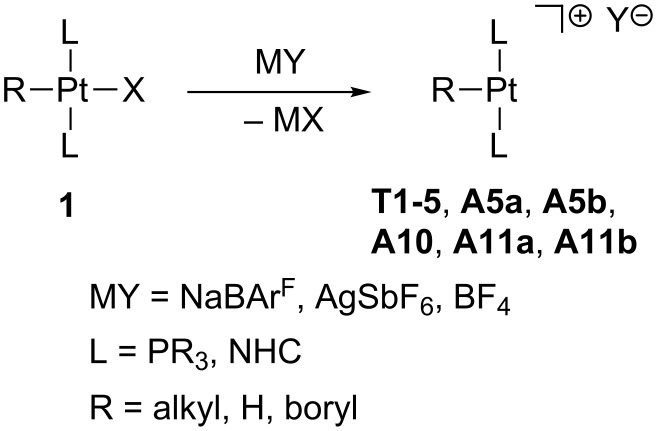
Halogen abstraction from **1**.

Several methods have been described to prepare coordinatively unsaturated Pt(II) complexes with a T-shaped geometry. Although the number of species that have been authenticated by crystallographic or spectroscopic methods is still very limited, the best and most general method for obtaining them is by removing a halogen ligand (Cl, Br, I) from the platinum coordination center in **1** by using a halogen abstractor with a poor coordinating anion, such as tetrakis[3,5-(trifluoromethyl)phenyl]borate or hexafluoroantimonate (Scheme 1) [19–21,28,39,44].

**Scheme 2:**
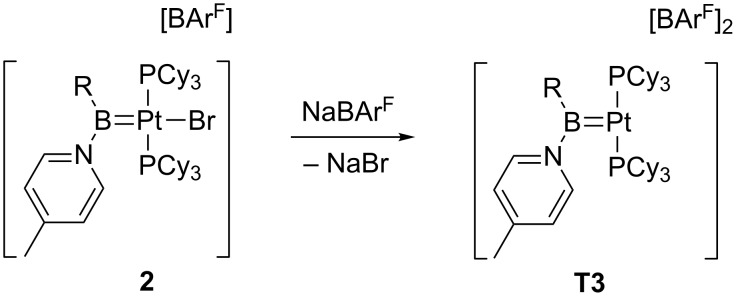
Halogen abstraction from **2** forming the dicationic complex **T3** [22].

This procedure has been successfully employed for the generation of complexes stabilized by two phosphine PR_3_ or two NHC ligands, in which the third coordination site is occupied by alkyl, hydride or boryl ligands. Therefore, high *trans*-influence ligands are present in all these cases, favoring the dissociation of the Pt–X bond. The generality of this method is so powerful that it has provided access to an intriguing dicationic borylene Pt(II) complex **T3** which is not stabilized by agostic interactions (Scheme 2) [22].

A stable T-shaped structure *trans*-[Pt(Me)(PiPr_3_)_2_]^+^ (**A7**) has also been generated [41] by methide abstraction from the neutral derivative *cis*-[Pt(Me)_2_(PiPr_3_)_2_] by using highly electrophilic Lewis acids such as B(C_6_F_5_)_3_ or [CPh_3_][1-H-*closo*-CB_11_Me_11_] (with the concomitant formation of MeB(C_6_F_5_)_3_^−^ or MeCPh_3_, respectively). This synthetic route uses the same procedure described by Goldberg et al. for the generation of a transient, neutral Pt(II) derivative [Pt(Me)(κ^2^-Tp^Me2^)] from the anionic Pt(II) complex K[Pt(Me)_2_(κ^2^-Tp^Me2^)] [95]. Alternatively, the cationic compound [Pt(Me)(PiPr_3_)_2_]^+^ was synthesized by homolytic cleavage of the Pt–Me bond by using the neutral radical [1-H-*closo*-CB_11_Me_11_]^•^ as a reagent.

**Scheme 3:**
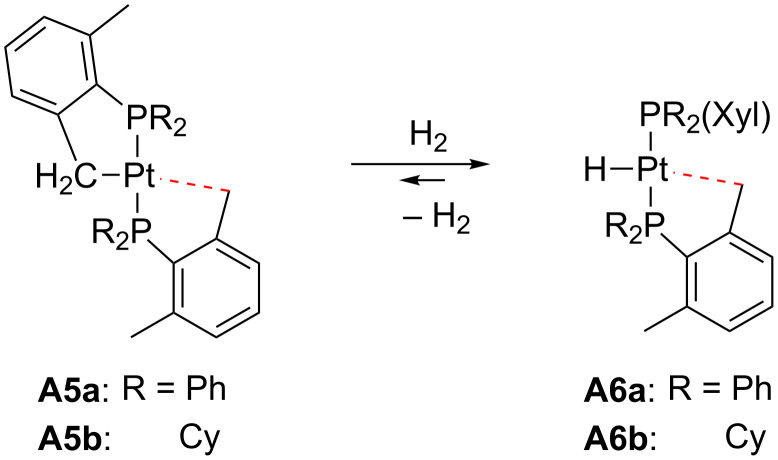
Hydrogenation of complexes **A5a** and **A5b** [39].

H_2_ addition across Pt–C_alkyl_ bonds can also lead to electron-deficient Pt(II) species stabilized by agostic interactions. Baratta and co-workers studied the addition of H_2_ to preformed T-shaped Pt(II) complexes **A5a**,**b** to prepare Pt(II) hydrides **A6a**,**b** (Scheme 3) [39].

**Scheme 4:**
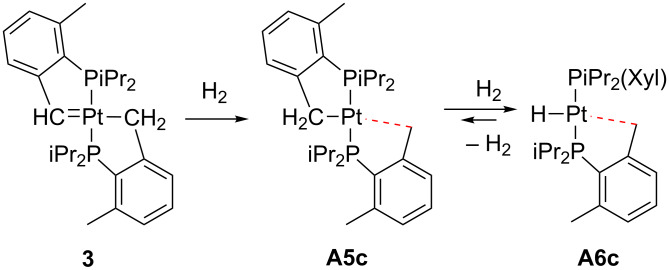
Hydrogenation of complexes **3** and **A5c** [40].

Alternatively, Carmona and co-workers have recently described that the hydrogenation of the 16-electron Pt(II) carbene **3** bearing a cyclometalated phosphine ligand (Scheme 4) resulted in the formation of the cyclometalated complex **A5c**, which can be further hydrogenated to give the Pt(II) hydride **A6c** [40], similar to those reported by Baratta [39].

**Scheme 5:**
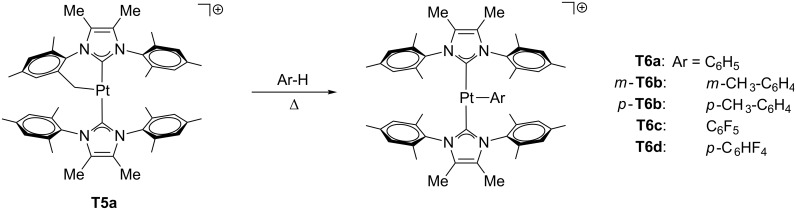
Intermolecular C–H bond activation from **T5a** [28].

Low electron-count species can also be prepared by C–H bond-activation reactions from coordinatively unsaturated compounds in a way similar to the addition of H_2_ to Pt–C_alkyl_ bonds mentioned above. The cyclometalated complex **T5a** shown in Scheme 5 reacts with aromatic compounds to yield the corresponding aryl complexes while keeping the unsaturated nature at the platinum atom. The complexes thus synthesized are not stabilized by agostic interactions according to spectroscopic (NMR), crystallographic and theoretical methods [28].

**Scheme 6:**
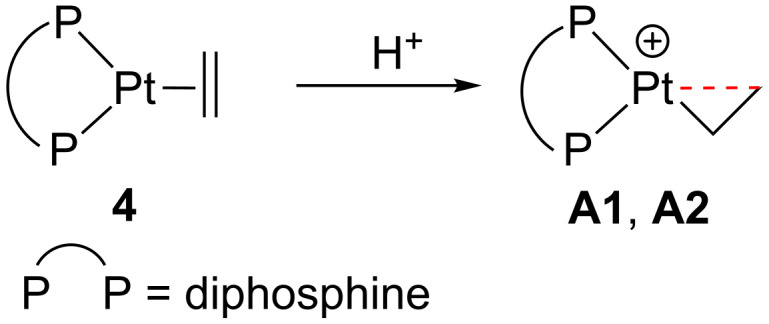
Protonation of complexes **4** [35–36].

Spencer et al. reported that the formation of stable electron-deficient Pt(II) complexes stabilized by strong β-agostic interactions can be accessible by protonation of electron-rich alkene Pt(0) compounds **4** (Scheme 6) [35–36].

**Scheme 7:**
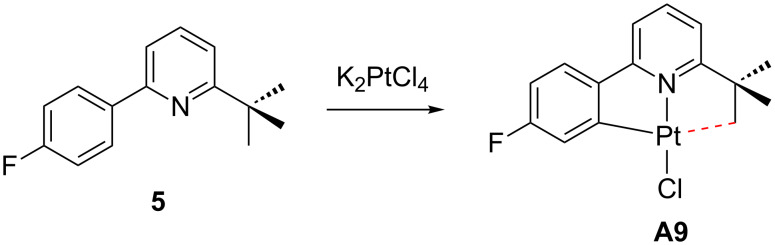
Cyclometalation of **5** [43].

Finally, Rourke et al. isolated a neutral Pt(II) derivative **A9** by a direct and simple approach that involves a cyclometalation process of the 2-substituted bulky pyridine **5** when it is reacted with the platinum salt K_2_PtCl_4_ (Scheme 7) [43].

#### Pt(II) complexes stabilized by solvent molecules

**Scheme 8:**
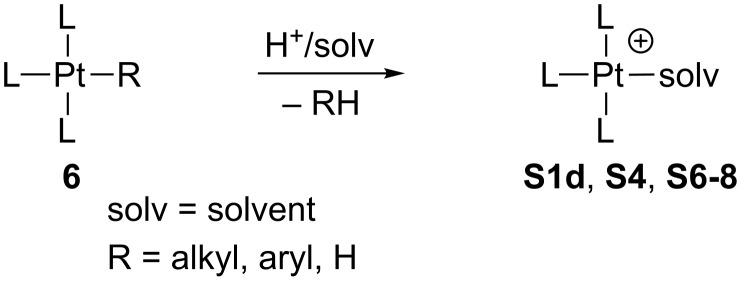
Protonation of **6**.

Although some of these compounds have been prepared by halogen [58] or methide [74] abstraction as described above, the vast majority of the Pt(II) derivatives stabilized by solvent molecules have been obtained by protonation of neutral alkyl, aryl or hydride Pt(II), as shown in Scheme 8 (see for example: [55,59–60,63,67,69,72,77–81]), or Pt(IV) complexes [96–98]. Nonetheless, this method has not yet been exploited for the preparation of true 14-electron species or agostic stabilized derivatives.

#### Transient electron-deficient Pt(II) complexes from six- and five-coordinate Pt(IV) derivatives

**Scheme 9:**
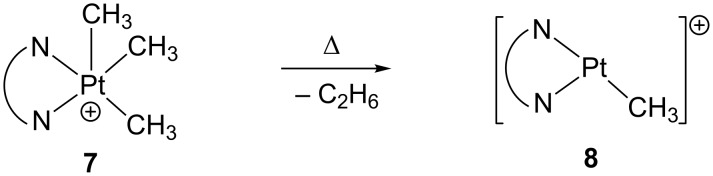
Reductive elimination of ethane from **7**.

In some cases, transient, highly reactive 14-electron Pt(II) compounds have been generated by thermal decomposition of trimethyl, five-coordinate Pt(IV) complexes **7**. These latter compounds are in some cases sufficiently stable to be isolated and characterized by X-ray diffraction studies, but thermally unstable at moderate to high temperatures releasing ethane and a three-coordinate Pt(II) intermediate [Pt(Me)L_2_]^+^ (Scheme 9). The coordinatively unsaturated complexes [Pt(Me)L_2_]^+^ thus generated undergo subsequent reactivity with appropriate reagents [99–102].

**Scheme 10:**
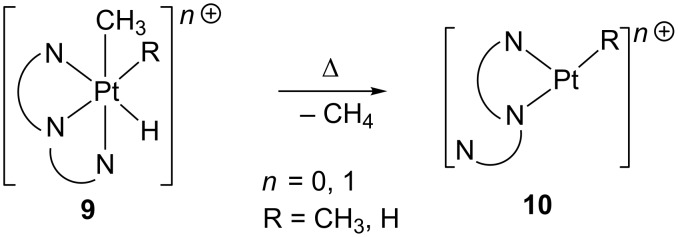
Reductive elimination of methane from six-coordinate Pt(IV) complexes.

Similarly, methane reductive elimination from Pt(IV) complexes [PtH(Me)(R)(η^3^-N_3_)]*^n^*^+^ **9** (R = Me, H; N_3_ = tris-pyridine[2.1.1]-(2,6)-pyridinophane or tris(pyrazolyl)borate; *n* = 0, 1) produces a transient three-coordinate Pt(II) complex [Pt(R)(η^2^-N_3_)]*^n^*^+^ **10** (Scheme 10), which is able to activate C–H bonds of hydrocarbons or form solvent adducts [103–105].

### Solution behavior

In masked three-coordinate d^8^ complexes, the fourth coordination site is occupied by a weakly bound ligand, which can be easily displaced. In this way, in solution the ligands of these compounds are opened to conformational events that will be discussed in this section. The empty site can also be available for incoming substrates, allowing the participation of these complexes as reaction intermediates. This aspect will be discussed in the next section.

Most of the reports of fluxional processes correspond to agostic bonded complexes. The equivalence of ^1^H and ^13^C chemical shifts observed in solution for the γ-, δ- and remote-agostic contacts is a common feature of the complexes collected in Figure 15 and arises from the rapid intramolecular exchange of the C–H bond involved in the agostic interaction. In several cases, such as **A11a** [44], the overlapping of the proton signals and signal averaging of the ligands avoid drawing a conclusion about the nature of the agostic interaction at work in solution. Recently, ab initio molecular dynamics (AIMD) simulations of some representative T-shaped Pt(II) complexes (**T5b**, **A2b** and **A11a**), performed in explicit dichloromethane solvent, have provided a detailed description of the mechanism by which the equivalence of signals takes place [106]. Simulations showed that the dynamics of the agostic interaction in solution strongly depends on the complex. Contingent upon the strength of the agostic interaction and the flexibility of the ligand, several events related with the occupation of the fourth coordination site by an agostic bond could happen: (i) the same C–H bond maintains the agostic interaction with the platinum atom for the entire simulation (**A2b**); (ii) an agostic interaction is present throughout the simulation, though the C–H bond changes by a rotation of alkyl groups (**A11b**); (iii) the agostic interaction moves on and off (**A11a**) [106].

There are indirect evidences that agostic bonds, solvent and weakly coordinating counteranions could easily exchange their role of stabilizing the unsaturated complex by placement at the vacant site. Although no kinetic studies on exchanges of these kinds of ligands have been reported, the detection of the agostic and solvent complexes for the same system and, for pincer complexes, of the counteranion and solvent forms, suggests that the exchange can take place. For instance, in *trans*-[Pt(Me)(PiPr_3_)_2_]^+^ (**A7**) a γ-agostic interaction can be displaced by addition of THF, forming the corresponding adduct [41]. NMR data in CD_3_CN of the triflate complex [Pt(BQA)(OTf)] (**C4a**) leads to its formulation as [Pt(BQA)(NCCD_3_)][OTf] (**S12**) [50]. Similar behavior has been observed for other pincer complexes. The naphthyl-based PCP–Pt(II) complex was found in THF as a 1:2 mixture of the counteranion (BF_4_^−^, **C8**) and solvent (**S17**) forms [53].

**Scheme 11:**
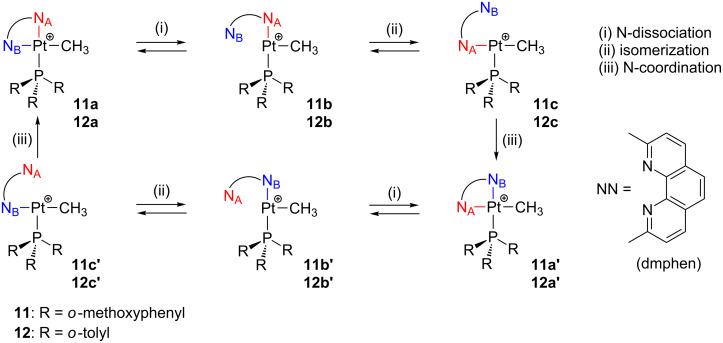
Proposed dissociative mechanism for the fluxional motion of dmphen in [Pt(Me)(dmphen)(PR_3_)]^+^ complexes.

Energetically accessible T-shaped species can also be intermediates in the site exchange of bidentate ligands of square-planar complexes. Fluxional motions of the 2,9-dimethyl-1,10-phenanthroline ligand (dmphen) have been observed in cationic complexes as [Pt(Me)(NN)(L)]^+^ (NN = dmphen; L = SOR(R’), PR_3_) [107–111]. The driving force of these flipping processes is the distortion of the dmphen ligand with respect to the coordination plane, which is caused by the methyl groups. Indeed, the fluxional motion is not detected when the unhindered 1,10-phenanthroline ligand is used. Interestingly, the mechanism can be switchable between associative and dissociative [107–109]. For the latter scenario, 14-electron T-shaped species involving phosphine ligands can be envisaged as feasible intermediates. The dissociative pathway fully prevails when overcrowded PR_3_ ligands are employed, where R stands for *o*-methoxyphenyl **11** [110] and *o*-tolyl **12** [111]. The detailed mechanism (Scheme 11) is described as follows: (i) dissociative nitrogen decomplexation, (ii) isomerization between two nonequivalent exchanging sites, and (iii) nitrogen coordination recovering the initial chelating situation. The fluxional motion of dmphen is not affected by the counteranion, solvent, or the presence of weak nucleophiles deliberately added. In addition, for **11a** and **12a** both flipping of dmphen and phosphine rotation motions are synchronized, behaving as molecular gears. The calculated activation barriers are ca. 13.3 and 16.4 kcal mol^−1^ for the motions involving **11** and **12**, respectively.

**Figure 16:**
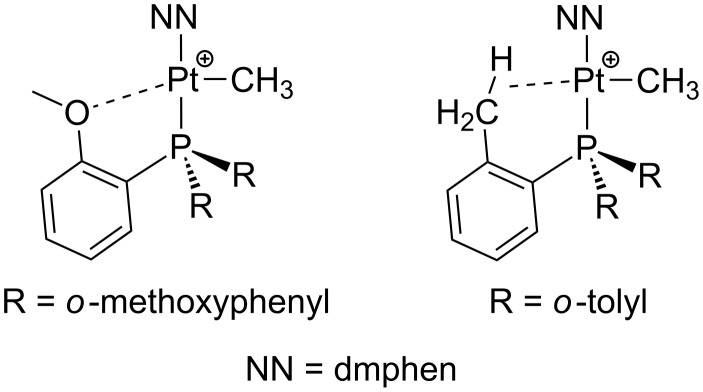
Feasible interactions for unsaturated intermediates **11b** (left) and **12b** (right) during fluxional motions [110–111].

Concerning 14-electron intermediates **b** and **b’**, the assistance of methoxy groups in **11b**,**b’** [110] as well as the presence of agostic interactions in **12b**,**b’** [111] can be postulated (Figure 16). Indeed, **12a** easily undergoes a cyclometalation process, probably assisted by an agostic contact via intermediate **12b**.

### Three-coordinate Pt(II) species as reaction intermediates

Coordinatively unsaturated Pt(II) species are considered as intermediates in many organometallic processes. Early studies by Whitesides and co-workers dealing with the thermal decomposition of [Pt(R)_2_L_2_] complexes demonstrated that the dissociation of a phosphine ligand is a preliminary requisite for the reaction to occur [112]. Since then, T-shaped three-coordinate 14-electron intermediates have been proposed in a large number of reaction mechanisms. A review concerning dissociative pathways in Pt(II) complexes was published in 1990 [113].

Due to both the low electron count and the presence of a vacant site in the coordination sphere, T-shaped Pt(II) species are suitable intermediates in ligand-exchange and bond-breaking processes of unreactive bonds, such as C–H bond activations. A selection of results from the past few years regarding these two issues are collected in this section to illustrate the growing importance of three-coordinate Pt(II) species as reaction intermediates.

### Intramolecular C–H bond activation

Intramolecular C–H bond activation is a common reaction of unsaturated Pt(II) complexes. Cyclometalation processes, sometimes involving agostic situations [114], have been thoroughly reviewed recently [115].

**Scheme 12:**
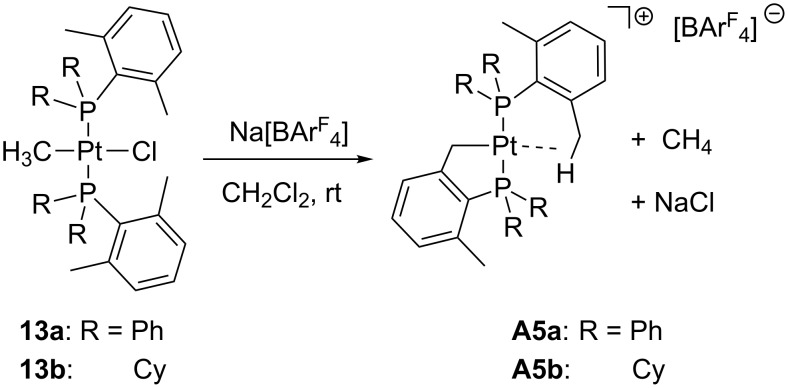
Halogen abstraction from **13a**,**b** and subsequent cyclometalation to yield complexes **A5a**,**b** [39].

According to Baratta and co-workers, the abstraction of Cl from **13a**,**b** with Na[BAr^F^] directly generates the cyclometalated products **A5a**,**b** and methane (Scheme 12) [39]. Unsaturated intermediates such as [Pt(Me)(PR_3_)_2_]^+^, probably stabilized by agostic contacts, might be considered.

**Scheme 13:**
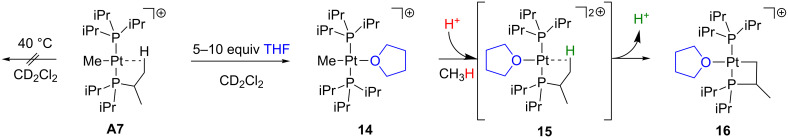
Proposed mechanism for the acid-catalyzed cyclometalation of **14** via intermediate **15** [41].

Weller et al. reported some reactivity features of complex **A7** [41]. Although it shows an agostic interaction, no cyclometalation via C–H bond activation is observed in CD_2_Cl_2_ (40 °C, 7 days). However, the addition of THF rapidly traps the T-shaped complex forming the adduct **14** (Scheme 13). This species does undergo cyclometalation in the presence of trace amounts of acids to yield **16** [116]. The unsaturated agostic species **15** has been proposed as a reaction intermediate.

**Scheme 14:**
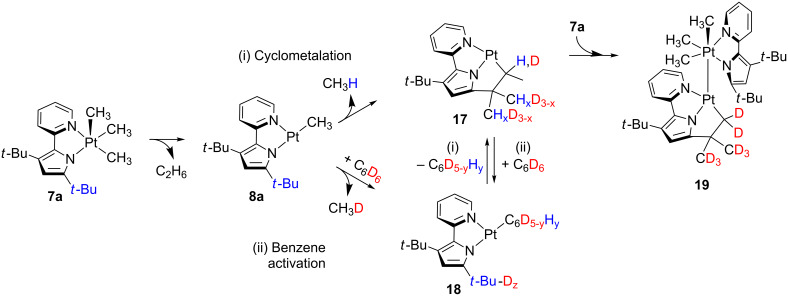
Proposed mechanism for the formation of **19** [102].

Goldberg and co-workers put forward the participation of T-shaped Pt(II) species in processes, implying the five-coordinate Pt(IV) complex **7a** (Scheme 14) [102]. This compound undergoes reductive elimination in benzene-*d*_6_ solvent with the concomitant formation of ethane. The resulting three-coordinate **8a** is supposed to be stabilized by an agostic interaction or solvent coordination. Indeed, two possible scenarios related to these interactions are envisaged. Firstly, **8a** evolves to **17** through a cyclometalation process ((i) in Scheme 14) which involves one *t-*Bu group of the bidentate ligand. On the other hand, **8a** can also activate the C–D bonds of benzene-*d*_6_ molecules forming **18** ((ii) in Scheme 14). Since both **17** and **18** exhibit potential empty coordination sites, benzene activation from **17** and cyclometalation from **18** are plausible scenarios. Nevertheless, the three-coordinate intermediate **17** is not isolable and reacts further with a molecule of the starting material **7a** leading to the dinuclear species **19**. The inclusion of deuterium atoms in the *t*-Bu groups of **19** suggests that both intra- and intermolecular C–H bond activations are competitive pathways. The transient formation of derivative **17** was confirmed by trapping experiments with ethylene.

**Scheme 15:**
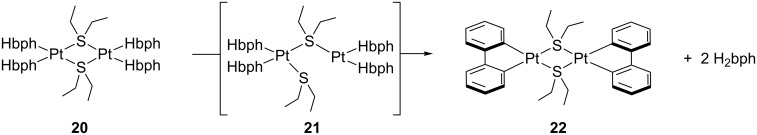
Cyclometalation of **20** via thioether dissociation [117].

Examples of cyclometalation processes preceded by ligand dissociation have been described by Romeo and co-workers [111,117–118]. For instance, [Pt_2_(Hbph)_4_(μ-SEt_2_)_2_] **20** (Hbph = η^1^-biphenyl monoanion) undergoes cyclometalation to form [Pt_2_(bph)_2_(μ-SEt_2_)_2_] **22** and biphenyl H_2_bph (Scheme 15) [117]. Intramolecular C–H bond activation seems to be driven by thioether dissociation via **21**, a process previously reported for the complex [Pt_2_(Me)_4_(μ-SMe_2_)_2_] [119].

**Figure 17:**
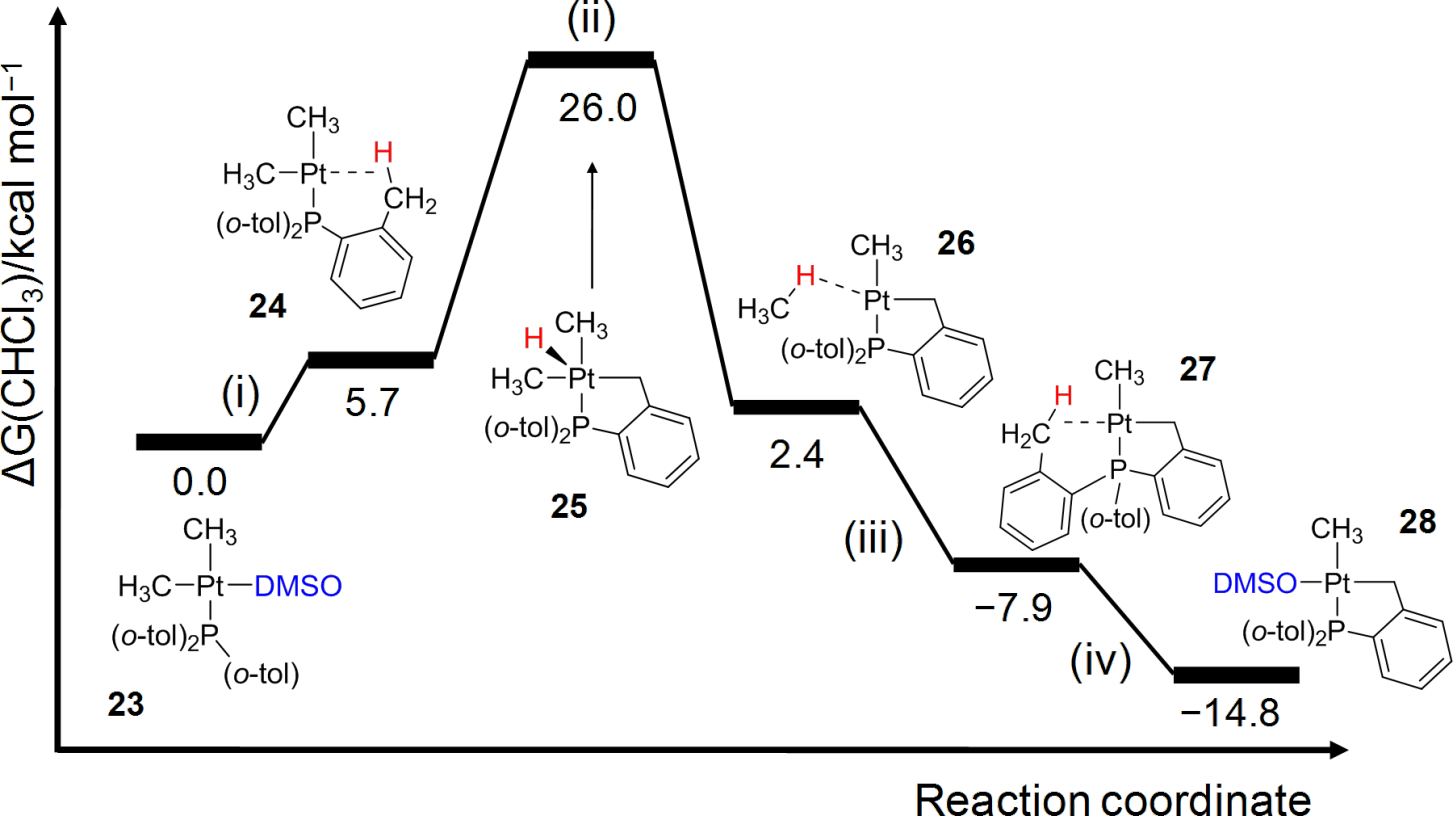
Gibbs energy profile (in chloroform solvent) for the cyclometalation of **23** [120].

Marrone et al. computed the cyclometalation process of [Pt(Me)_2_(PR_3_)(DMSO)] **23** to yield [Pt(Me)(PR_2_R’)(DMSO)] **28** (R = *o*-tolyl, R’ = cyclometalated group; DMSO = dimethylsulfoxide) [120] as experimentally reported in [118]. They demonstrated that the reaction involves coordinatively unsaturated 14-electron T-shaped complexes through (i) DMSO dissociation, (ii) C–H bond activation, (iii) methane release, and (iv) DMSO association (Figure 17). Starting from **23**, initial ligand dissociation generates the T-shaped intermediate **24**, in which one *o*-tolyl group of the phosphine ligand establishes an agostic interaction with the platinum center. This agostic interaction weakens the C–H bond inducing an oxidative-addition (OA) process. The resulting five-coordinate Pt(IV) hydride complex **25**, located at 26.0 kcal mol^−1^ above reactants, quickly undergoes reductive elimination (RE) providing the methane adduct **26**. After methane releasing, the cyclometalated T-shaped structure **27** is obtained, once again stabilized by an agostic interaction. The corresponding transition states for OA and RE processes are isoenergetic with respect to **25**. Finally, the agostic coordination mode in **27** is displaced by a DMSO molecule forming the cyclometalated product **28**. Moreover, they showed that the reaction mechanism starting from the four-coordinate 16-electron complex **23** does not provide low-energy paths for OA, demonstrating the kinetic inertness of these types of compounds.

**Scheme 16:**
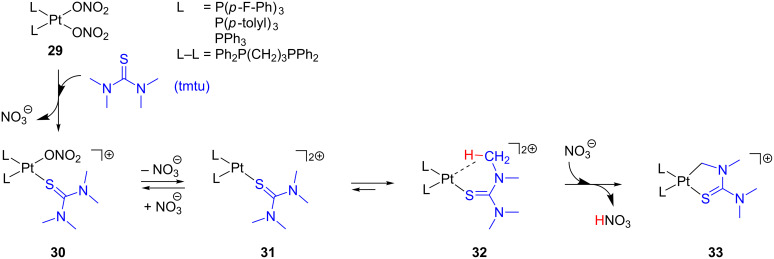
Coordination of tmtu to **29** and subsequent C–H bond activation via three-coordinate species **31** and **32** [121].

Three-coordinate species have been invoked by Nolan and co-workers to account for the reaction of *cis*-[Pt(NO_3_)_2_L_2_] **29** with tetramethylthiourea (tmtu) (Scheme 16) [121]. The sulfur atom of tmtu displaces one nitrate ligand of **29** forming **30**, which eventually evolves to the cyclometalated product **33** via C–H bond activation. The increase of nitrate concentration as well as the addition of coordinating agents, such as triethylamine and triphenylphosphine, clearly retard the process. Therefore, a dissociative mechanism has been proposed to generate the unsaturated species **31**. Then, one methyl group of the coordinated tmtu can stabilize the open coordination site via agostic interaction, **32**, inducing an intramolecular C–H bond activation process to yield **33**. It was also observed that less σ-donor phosphine ligands increase the reaction rate. Less-electron-donating ligands may not stabilize **31**, favoring an agostic contact in **32**, which, consequently, accelerates the cyclometalation reaction. Indeed, the presence of stronger σ-donors such as ICy ligand (1,3-biscyclohexylimidazol-2-ylidene) inhibits the process.

**Scheme 17:**
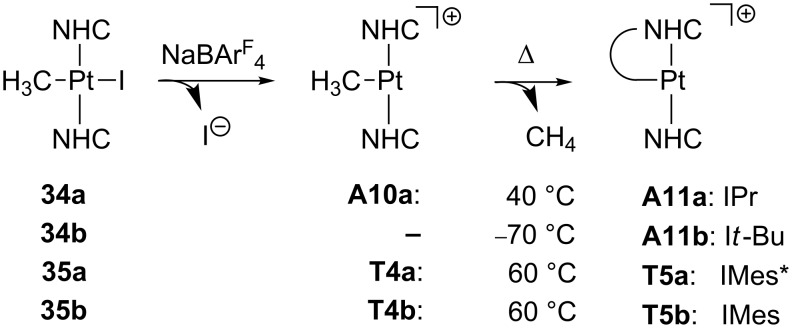
Cyclometalation process of NHC-based Pt(II) complexes [28,44].

T-shaped Pt(II) complexes bearing NHC ligands can be prepared starting from the pertinent iodo-precursors by halide removal (Scheme 17) [44]. For IPr [44], IMes* and IMes ligands [28], the corresponding methyl complexes **A10a** and **T4a**,**b** can be isolated. Interestingly, cyclometalation involving a methyl group of the carbene arm was observed upon heating these methyl derivatives. It is noteworthy that the process barely depends on the nature of the fourth coordination site, either agostic **A10a** or pure empty site **T4a**,**b**. In the case of I*t*-Bu ligand [44], a similar cyclometalation reaction is observed even at low temperatures (−70 °C), but the putative methyl intermediate could not be detected.

**Scheme 18:**
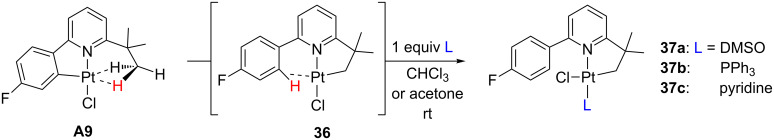
Cyclometalation process of complex **A9** [43].

The agostic complex **A9** can exchange the site of cyclometalation by C(sp^3^)–H bond activation (Scheme 18) [43]. The addition of one equivalent of L (L = DMSO, PPh_3_ or pyridine) to **A9** in chloroform or acetone at room temperature yields the products **37**, in which one methyl group (previously agostic) has been cyclometalated, and L has entered into the coordination sphere of the platinum atom. The authors reasonably propose a σ-bond metathesis (σ-CAM) mechanism via intermediate **36** invoking the well-known capacity of agostic interactions to facilitate the C–H bond cleavage.

**Scheme 19:**
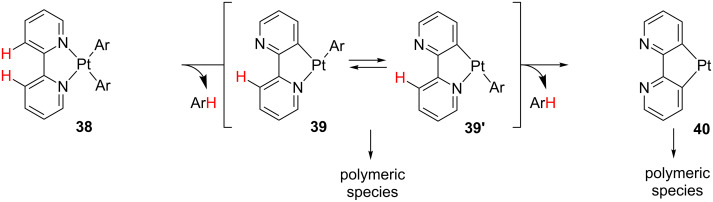
“Rollover” reaction of **38** and subsequent oligomerization [123].

The “rollover” process is a class of cyclometalation, in which a heteroaryl ligand undergoes decoordination and bond-rotation processes prior to C–H bond activation. A recent review on this topic collects the most important features of this reaction [122]. Early work by Young and co-workers [123] proposes the formation of coordinately unsaturated species **39** and **39’** as intermediates in the “rollover” reactions of **38**, leading to polymeric species (Scheme 19).

**Scheme 20:**
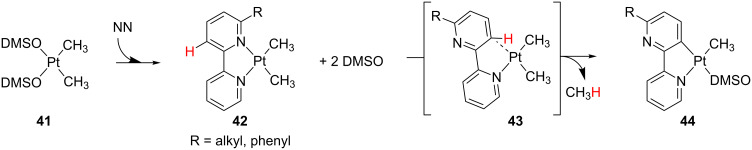
Proposed mechanism for the formation of cyclometalated species **44** [124].

Zucca and co-workers reported the synthesis of several cyclometalated compounds **44** by means of substitution and “rollover” processes (Scheme 20) [124]. Starting from derivative **41**, DMSO displacement by 6-substituted 2,2’-bipyridines (NN ligands) yields the corresponding bidentate derivatives **42**. Due to the steric congestion between the R group of the bipyridine and the methyl ligand **42** becomes unstable promoting the decoordination of the nitrogen atom. Subsequent C–H bond activation is proposed to take place through an agostic intermediate **43** generated by rotation around the 2,2’-C–C bond of the bipyridine ligand. After the release of methane, the vacant site is easily occupied by one DMSO ligand yielding **44**. Interestingly, from **44** (R = *t*-Bu, Ph) the corresponding hydride compounds can be prepared [125]. It is worth pointing out that, depending on the ligand present in solution, both 16-electron and 14-electron species are obtained, though the latter are not stable, and only oligomers with bridging hydrides can be detected [125].

**Scheme 21:**
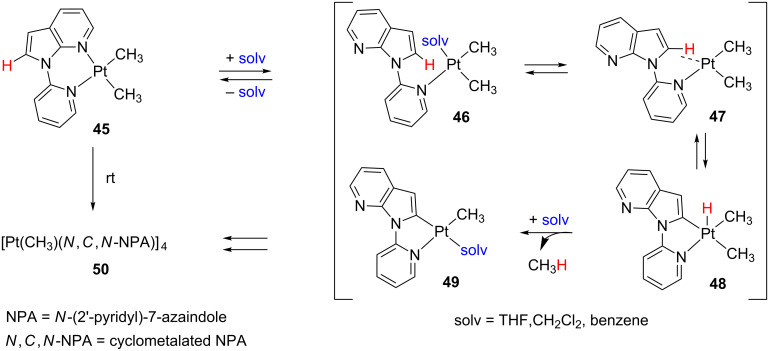
Self-assembling process of **45** by “rollover” reaction [126].

Wang and co-workers disclosed the spontaneous self-assembling of [Pt(Me)_2_(NPA)] **45** by a “rollover” cyclometalation process (Scheme 21) [126]. The suggested mechanism begins with a C–N bond rotation by chelating ligand dissociation forming species **46**, which is stabilized by solvent coordination. Eventually, an agostic interaction in **47** prior to the rate-determining C–H bond cleavage should displace the solvent molecule. An oxidative addition and reductive elimination (OA/RE) scenario via hydride Pt(IV) **48** and subsequent methane release yield the corresponding solvent adduct **49**, from which self-association generates the cyclic tetramer **50**. Indeed, when good coordinating agents such as acetonitrile are added, the reaction slows down.

**Scheme 22:**
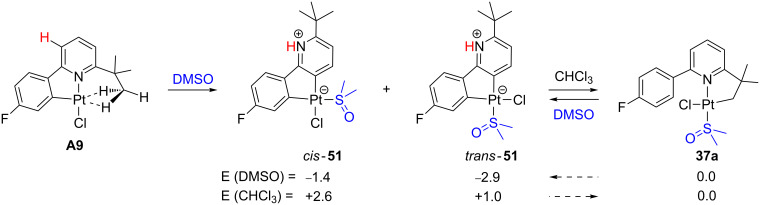
“Rollover” reaction of **A9**. Energies (solvent) in kcal mol^−1^ [127].

A “rollover” process has been observed for the already cyclometalated compound **A9** in DMSO providing two zwitterionic products, *cis*- and *trans*-**51** (Scheme 22) [127]. On the other hand, by switching the DMSO solvent to the less polar chloroform the expected cyclometalated product **37a** (Scheme 18) is obtained. DFT calculations correctly explain the relative stabilities of **51** with respect to **37a** depending on the polarity of the solvent [127].

**Scheme 23:**
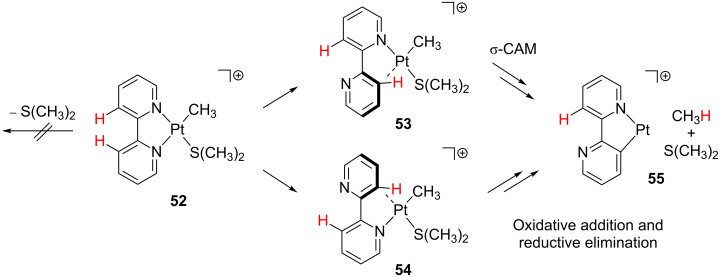
Proposed mechanisms for the “rollover” cyclometalation of **52** in gas-phase ion-molecule reactions [128].

This kind of reactivity has also attracted attention in gas-phase conditions [122]. The gas-phase behavior of cationic species **52** has been analyzed by means of combined experimental and computational studies (Scheme 23) [128]. DFT calculations discourage the initial loss of dimethylsulfide. Instead, decomplexation and C–C bond rotation processes starting from the four-coordinate complex **52** are favored. The resulting isomeric compounds exhibit an empty coordination site that is filled by an agostic interaction prior to the C–H bond activation. Intermediate **53** evolves through a σ-CAM process, whereas intermediate **54** undergoes oxidative addition and reductive elimination processes. The release of methane and dimethylsulfide yields **55**. Further studies including labeling experiments support the reversibility of these “rollover” reactions. The highly unsaturated species **55** is still reactive and can coordinate and decompose XMe_2_ molecules (X = S [128] and O [129]) and dehydrogenate alkanes [130]. Finally, other cyclometalation processes including “rollover” reactions have also been observed for the complex [Pt(Me)L(SMe_2_)] bearing a diimine ligand instead of the ubiquitous bipyridyl backbone [131].

### β-H elimination

**Scheme 24:**
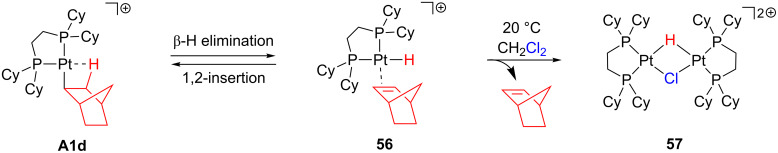
β-H elimination and 1,2-insertion equilibrium involving **A1d** and the subsequent generation of **57** [35].

As previously noted, the agostic complexes **A1**–**4** (Figure 7) are in equilibrium with the hydrido–alkene isomers [34–38]. Experimental evidence points out that substituted alkyls and large chelate ring-size diphosphine ligands favor the β-agostic isomer. As a representative example, Scheme 24 shows the β-H elimination for **A1d** together with the reverse 1,2-insertion for **56**. Interestingly, upon crystallization in dichloromethane solvent, **A1d** eliminates norbornene from **56** and generates the dinuclear complex **57** [35]. The presence of chloride as a bridging ligand suggests that solvent molecules are involved in the reaction. Therefore, the participation of T-shaped intermediates, probably stabilized as solvento adducts, might be relevant in the overall process.

**Scheme 25:**
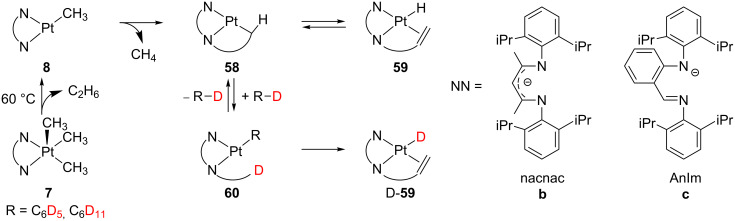
Proposed mechanism for thermolysis of **7b** and **7c** in benzene-*d*_6_ and cyclohexane-*d*_12_ solvents [101].

Although β-elimination should be easily accomplished, Goldberg and co-workers realized that other reactions can compete. The thermolysis of five-coordinate Pt(IV) complexes **7** containing nacnac ([{(*o*-iPr_2_C_6_H_3_)NC(CH_3_)}_2_CH]^−^, **7b**) [99,101] and AnIM ([*o*-C_6_H_4_-{N(C_6_H_3_iPr_2_)}(CH=NC_6_H_3_iPr_2_)]^−^, **7c**) [101] ligands produces D-**59** in benzene-*d*_6_ (Scheme 25). The first step in this reaction seems to be the direct reductive elimination of **7** liberating ethane. The resulting intermediate **8** undergoes cyclometalation to give the complex **58**. The subsequent β-H elimination process shall provide the expected hydride complex **59**. However, solvent molecules come into play, so that the unsaturated intermediate **58** activates the C–D bond of benzene-*d*_6_ forming **60**. Cyclometalation and subsequent β-H elimination processes generate D-**59**, which has fully incorporated the corresponding deuterium atoms. This latter evidence suggests that the intermolecular C–D bond activation of benzene-*d*_6_ is indeed faster than the β-elimination. On the other hand, when the reaction is conducted in a cyclohexane-*d*_12_ solution, D-**59** is hardly obtained and **59** prevails. It means that, in sharp contrast to the arene solvent, the intermolecular C–D bond activation of alkanes becomes slower than the β-elimination.

**Scheme 26:**
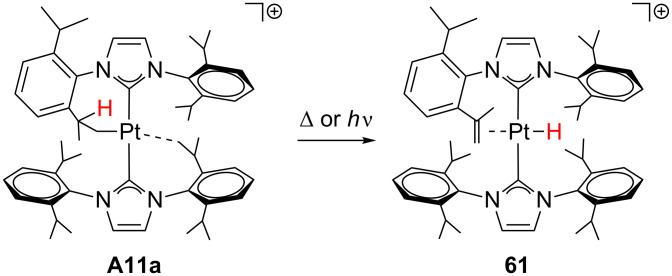
β-H elimination process of **A11a** [28].

This type of reaction has also been observed for the agostic complex **A11a** [28], although the CH group of the agostic contact is not involved. Upon heating or under UV irradiation (Scheme 26), one hydrogen of the cyclometalated isopropyl group in **A11a** undergoes a β-H elimination process yielding **61**, in which the alkene and the hydride ligands are located mutually *trans*.

### Intermolecular C–H bond activation

Three-coordinate T-shaped Pt(II) complexes have been postulated in hydrocarbon C–H bond activations, particularly in the Shilov system for the functionalization of methane. A number of excellent reviews about C–H bond activation have been published [132–137].

**Scheme 27:**
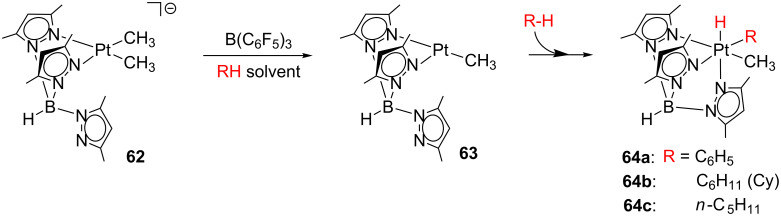
Intermolecular C–H bond activation from **62** [95].

Labile ligands in masked T-shaped compounds allow further reactivity. Although some solvent complexes exhibit associative pathways eluding 14-electron species [73,138], the participation of such coordinatively unsaturated intermediates should be taken into account for other systems [135–136]. A good example is the investigation reported by Wick and Goldberg (Scheme 27) [95]. From the anionic species [Pt(Me)_2_Tp’]^−^ **62**, they attempted to generate the unsaturated species **63** through the abstraction of one methyl ligand. Indeed, the treatment of **62** with B(C_6_F_5_)_3_ in benzene, cyclohexane and *n*-pentane provides the products **64** arising from the C–H bond activation of solvent molecules. Interestingly, the activation of pentane molecules only occurs at the primary carbon atom.

**Scheme 28:**
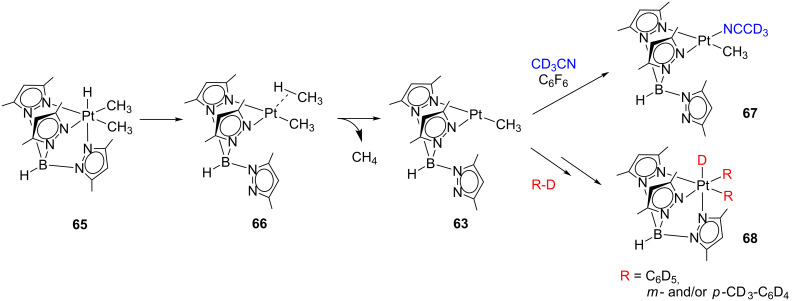
Reductive elimination of methane from **65** followed by CD_3_CN coordination or C–D bond-activation processes [105].

A similar situation has been observed for the reductive elimination of methane in the complex **65** (Scheme 28) [105]. Experimental evidence is consistent with a dissociative methane loss from **66** as the rate-determining step. Therefore, as shown in Scheme 27, the unsaturated intermediate **63** is supposed to operate again. When the reaction is carried out in CD_3_CN/C_6_F_6_ mixtures, acetonitrile binds the transient **63** forming the corresponding adduct **67**. On the other hand, when benzene-*d*_6_ and toluene-*d*_8_ are used as solvents, intermolecular C–D bond activations occur with the formation of **68**. In cyclohexane-*d*_12_ solution, the observation of deuterated methane isotopomers indicates C–D bond-activation processes, though the corresponding alkyl Pt(IV) product could not be characterized.

**Figure 18:**
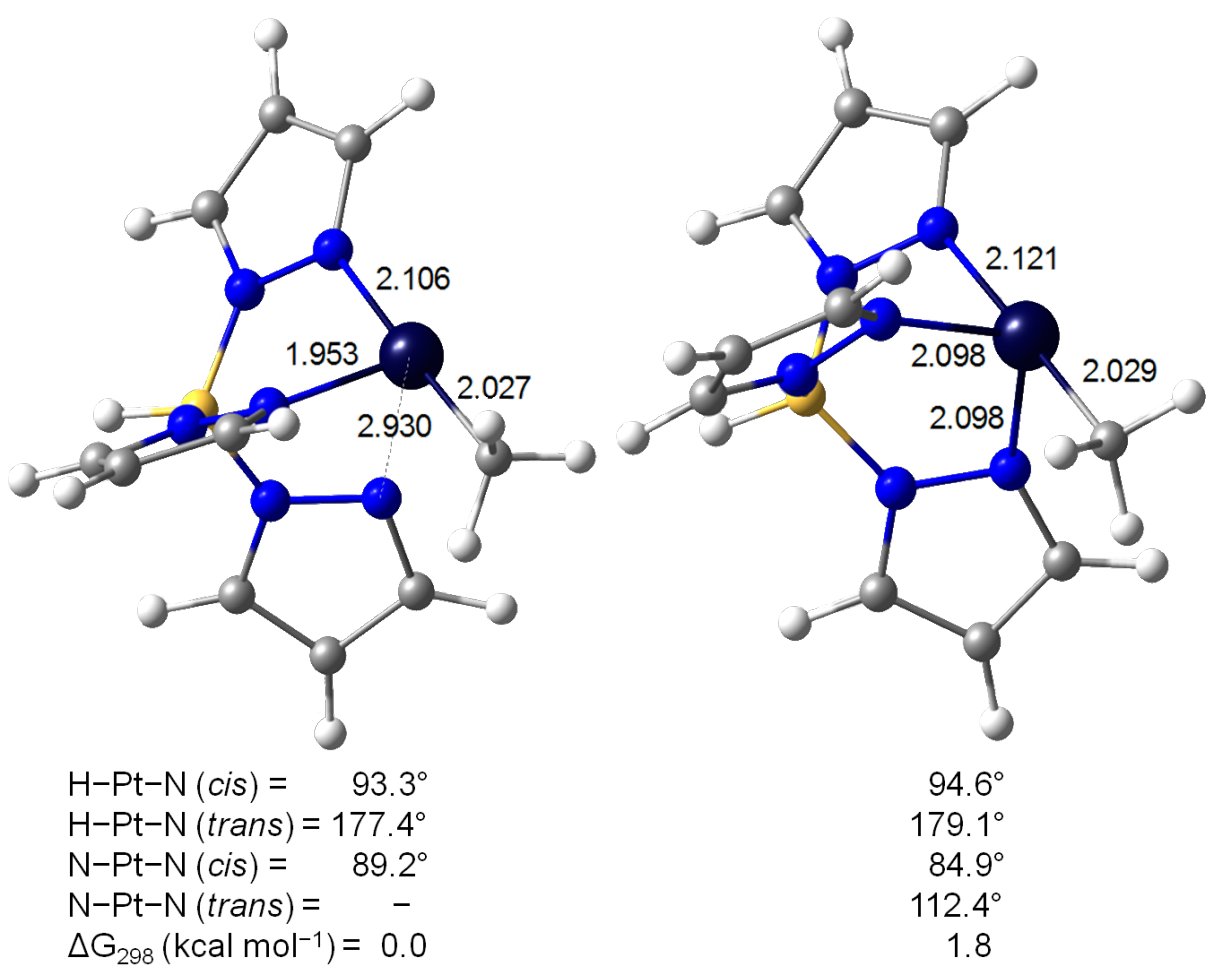
DFT-optimized structures describing the κ^2^ (**69**, left) and κ^3^ (**69’**, right) coordination modes of [Pt(Me)Tp] [139]. Bond distances in angstroms, angles in degrees and Gibbs energies in kcal mol^−1^.

Nevertheless, the participation of one arm of the Tp’ ligand stabilizing the open coordination site in **63** should be considered. As pointed out by Keinan and co-workers [139], the intermediate [Pt(Me)Tp] can adopt two different structures; the bidentate κ^2^ coordination mode (**69**) provides a T-shaped structure (Figure 18 left) whereas the κ^3^-complex (**69’**) exhibits a see-saw geometry (Figure 18 right). The former is slightly favored by only 1.8 kcal mol^−1^.

**Scheme 29:**
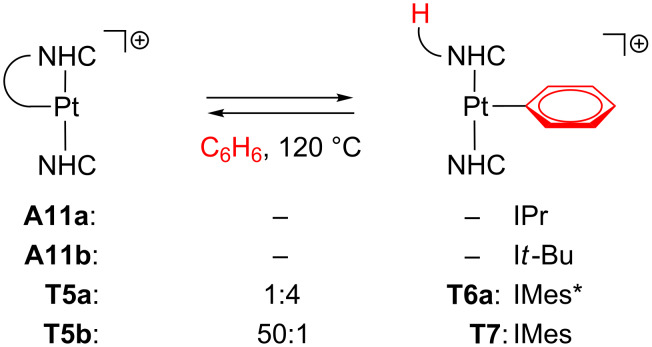
Intermolecular arene C–H bond activation from NHC-based complexes [28].

Recently, arene activation has been reported regarding true T-shaped species with NHC ligands [28]. The cyclometalated complexes **A11a**, **A11b**, **T5a** and **T5b** were tested toward C–H bond-activation processes by using benzene as a solvent (Scheme 29). No reaction was observed for **A11a** and **A11b** even under drastic conditions (high temperatures and long reaction times). On the other hand, **T5a** yields the phenyl product **T6a**, whereas **T5b** barely reacts.

**Figure 19:**
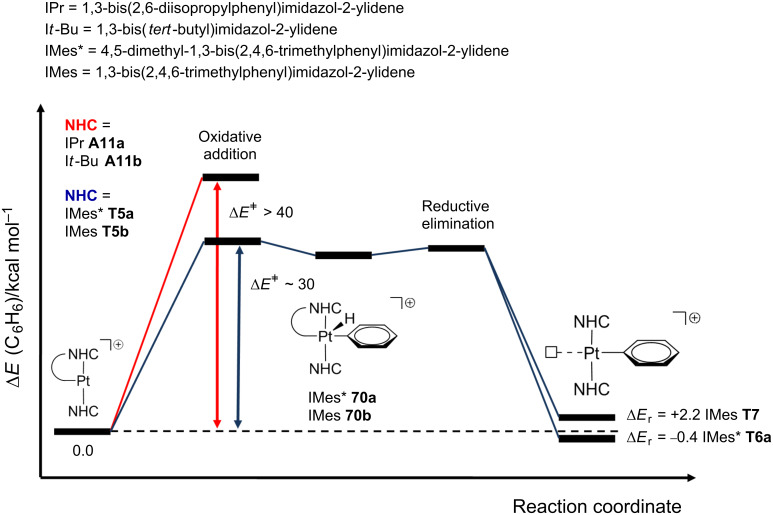
Energy profiles (in benzene solvent) for the benzene C–H bond activation from **A11a**, **A11b**, **T5a** and **T5b** [28].

DFT calculations suggest an oxidative addition and reductive elimination scenario via Pt(IV) hydride intermediates **70** (Figure 19). The steric environment of the agostic complexes **A11a** and **A11b** complicates the reaction, which is reflected in the high energy barriers (more than 40 kcal mol^−1^, red line). In sharp contrast, the true T-shaped species **T5a** and **T5b**, with no agostic bonds, show lower energy profiles (ca. 30 kcal mol^−1^, blue line) and the reaction thermodynamics (Δ*E*_r_ values, Figure 19) accounts for the observation of **T6a** and the poor detection of **T7**.

Other arenes can also be activated by the use of **T5a** to afford **T6** (Figure 6). In toluene solvent, only the two products *m*-**T6b** and *p*-**T6b** corresponding to *meta-* and *para*-site activations are observed in a 5:1 molar ratio. Neither *ortho*- nor benzylic C–H bond activations are detected. Once again, DFT calculations provide reasonable Δ*E*^‡^ for both *meta* and *para*-routes (ca. 30 kcal mol^−1^), and the thermodynamic effects, i.e., that only *m*-**T6b** and *p*-**T6b** are slightly more stable than **T5a**, explain the experimental evidence.

**Scheme 30:**
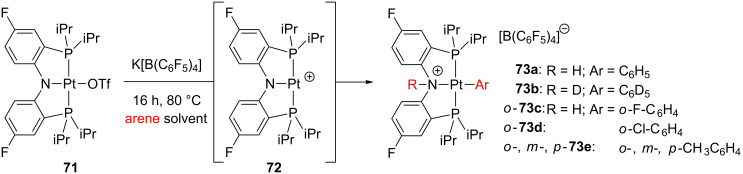
Intermolecular arene C–H bond activation from PNP-based complex **71** [12].

Some pincer complexes can indeed activate the C–H bonds of benzene, though the mechanism is not fully understood [50,140]. In this line, Ozerov and co-workers have attempted to access unsaturated species by means of abstraction of triflate ligand from **71** (Scheme 30) [12]. The presumable generation of the low electron-count intermediate **72**, results in the intermolecular C–H bond activation of several arene solvents. Phenyl **73a** and phenyl-*d*_5_ **73b** together with *o*-fluoro **73c** and *o*-chlorophenyl **73d** are obtained. The toluene solvent also reacts forming a mixture of *o*-, *m*- and *p*-tolyl complexes (8% *o*-**73e** and 92% *m*-**73e** and *p*-**73e**). Additional experiments proved that the reverse processes are not kinetically accessible. In some cases, C–X bond-activation processes involving PhBr and CH_2_Cl_2_ molecules have also been registered.

**Scheme 31:**
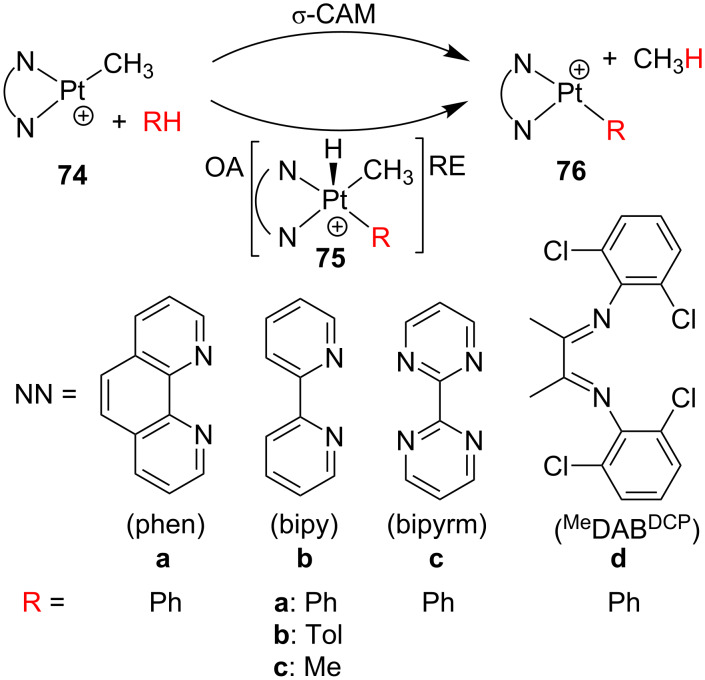
Intermolecular C–H bond-activation by gas-phase ion-molecule reactions of **74** [7,142].

Complexes of the type [Pt(Me)(NN)]^+^ **74** (Scheme 31) have shown reactivity toward the C–H bond activation of benzene in gas-phase ion-molecule reactions [7]. Suitable labeling experiments disclose the reversibility of the C–H bond activation. Interestingly, the relative rate constants reveal that **74d** reacts slower than **74a**–**c**. In the absence of solvent, both reactants **74a**–**c** and phenyl products **76a**–**c** are believed to exhibit true T-shaped structures. On the other hand, the lower reactivity of **74d** has been attributed to an interaction of the empty site of the platinum with one *ortho*-chlorine atom of the (*o*,*o*’-Cl_2_C_6_H_3_)N=C(CH_3_)–C(CH_3_)=N(*o*,*o*’-Cl_2_C_6_H_3_) (^Me^DAB^DCP^) ligand [141], which blocks the approach of a benzene molecule. Oxidative addition and reductive elimination (OA/RE) as well as σ-CAM scenarios have been evaluated for **74b**, but unfortunately the results are functional-dependent. B3LYP cannot reproduce the experimental evidences, mPW1K and mPW1PW91 favor the σ-CAM mechanism, and M05-2X considers both of them [7]. The bipyridyl complex **74b** can also activate toluene and methane molecules forming **74bb** and **74bc**, respectively [142]. Concerning the toluene activation, *meta*-, *para*- and benzylic positions can be activated forming *m*-**76bb**, *p*-**76bb** and Bn-**76bb**, respectively. BP86 calculations suggest that OA/RE and σ-CAM are competitive pathways.

### H_2_ activation

**Scheme 32:**
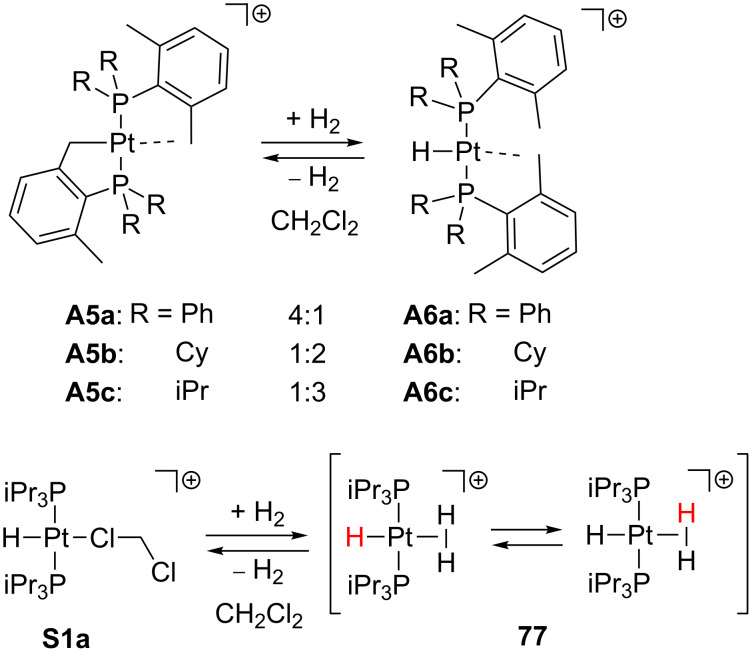
Dihydrogen activation through complexes **A5a**, **A5b** [39], **A5c** [40] and **S1a** [54].

Some agostic complexes previously reported can activate small molecules such as H_2_ (Scheme 32). For instance, the complexes **A5a** and **A5b** react with dihydrogen (1 atm H_2_, 20 °C) in dichloromethane solution to yield the corresponding agostic hydride products **A6a** and **A6b** [39]. Both species were found in equilibrium, exhibiting **A5**/**A6** ratios of 4:1 and 1:2 for species **a** and **b**, respectively. A similar behavior is observed for the analogous reaction of **A5c** (1 bar H_2_, −30 °C to room temperature), in which **A6c** is obtained in a 1:3 ratio [40]. These results suggest that hydride species seem to be favored according to the basicity of the phosphine ligand. In previous works, the dichloromethane molecule in the solvento complex **S1a** was labile enough to allow H_2_ activation but, unlike **A6**, the corresponding dihydrogen adduct **77** was observed (Scheme 32) [54]. The latter undergoes a fluxional process which can be slowed down by cooling at −60 °C.

**Scheme 33:**
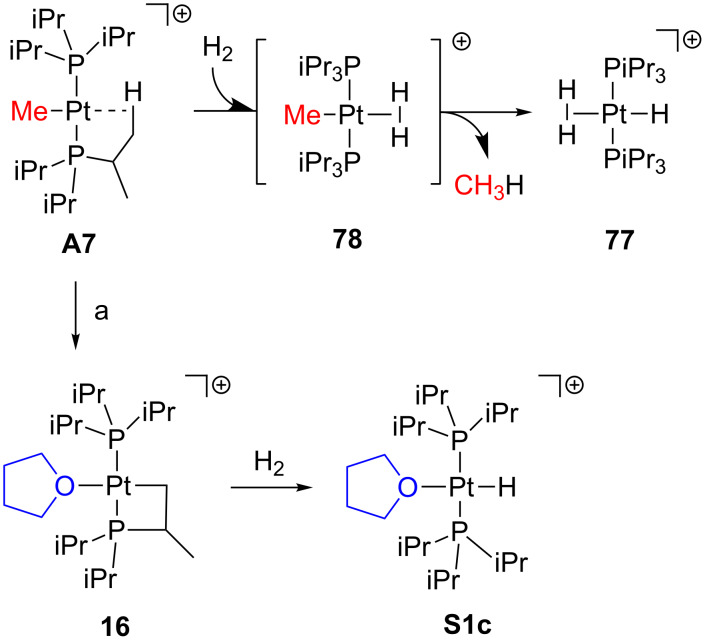
Dihydrogen activation through complexes **A7** and **16** [41]. For a: see Scheme 13.

Weller and co-workers reported an alternative procedure to obtain **77** from **A7** by addition of dihydrogen in CD_2_Cl_2_ solution (Scheme 33) [41]. The agostic interaction in **A7** is supposed to be displaced by one dihydrogen molecule forming the putative intermediate **78**. The concomitant release of methane may be the driving force of the reaction, thus the entropic factors together with the nature of the phosphine ligands should be taken into account. Additionally, the related compound **16** (Scheme 13) can also activate dihydrogen molecules; the cyclometalated ring is opened and the corresponding hydride complex **S1c** is obtained [54].

### X_2_ activation

**Scheme 34:**
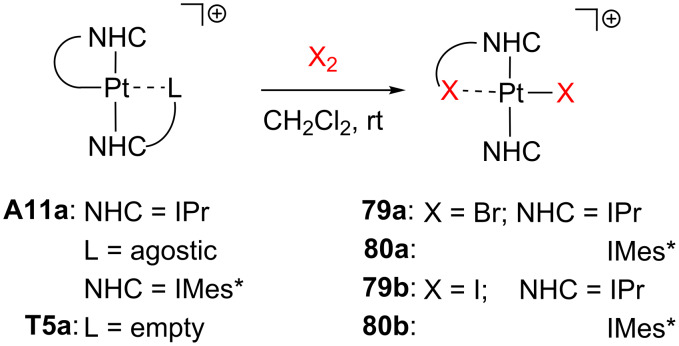
Br_2_ and I_2_ bond activations through complexes **A11a** and **T5a** [143].

The cyclometalated compounds **A11a** and **T5a** can activate X_2_ molecules (X = Br, I) in dichloromethane solution affording **79** and **80** in which one C(sp^3^)–X bond is constructed (Scheme 34) [143]. It is noteworthy that the reaction occurs in the presence (**A11a**) or the absence (**T5a**) of agostic interactions in the starting materials.

**Scheme 35:**
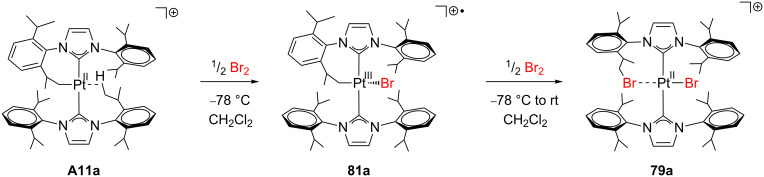
Detection and isolation of the Pt(III) complex **81a** [143].

Interestingly, during the reaction of **A11a** with Br_2_ at low temperature (−78 °C) a paramagnetic, see-saw Pt(III) alkyl intermediate **81a** could be isolated and characterized (Scheme 35), although the iodo-analogue **81b** could not. DFT calculations correctly predicted the feasibility of **81a** (−9.5 kcal mol^−1^ below reactants) and explained the nondetection of **81b** (+4.5 kcal mol^−1^ above reactants) in terms of higher relative energies.

**Scheme 36:**
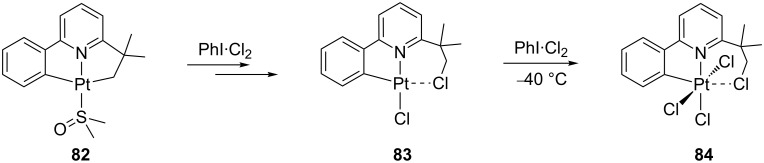
Cl_2_ bond activation through complexes **82** and **83** [144].

A related Cl_2_ activation process involving unsaturated species has been recently reported by Rourke and co-workers (Scheme 36) [144]. Under certain conditions, the reaction of **82** with PhI·Cl_2_ yields **83**. Although suitable crystals of **83** could not be obtained, the structure was elucidated by NMR studies showing the formation of one C(sp^3^)–Cl bond. Similarly to the agostic species **A9**, the resulting CH_2_Cl group is interacting with the open coordination site. Further reaction of **83** with PhI·Cl_2_ forms the oxidation product **84**.

### *cis*–*trans* Isomerization

**Scheme 37:**
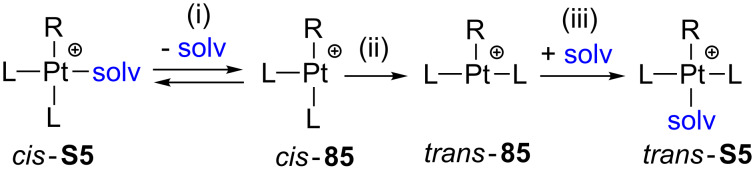
*cis*–*trans* Isomerization mechanism of the solvento Pt(II) complexes **S5** [2,61].

The *cis*–*trans* isomerization of several solvento species **S5** has been studied and the formation of low electron-count Pt(II) complexes **85** through dissociative pathways accounts for the experimental observations [2,61]. The general mechanism is depicted as follows: (i) rate-determining dissociation step of a solvent molecule, (ii) isomerization process, and (iii) fast association of a solvent molecule (Scheme 37).

**Figure 20:**
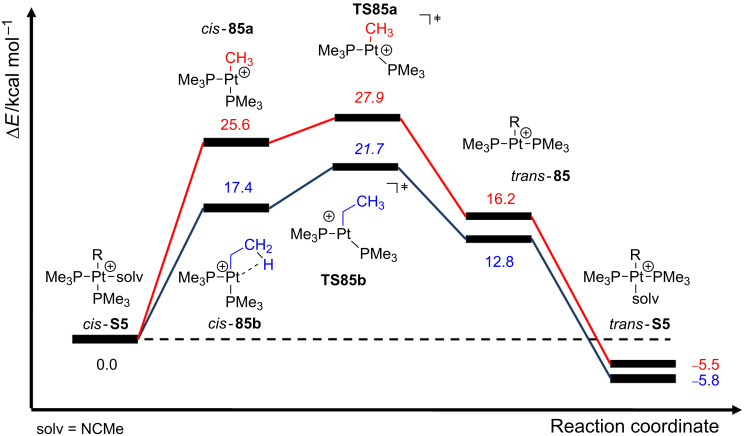
Energy profiles for the isomerization of complexes [Pt(R)(PMe_3_)_2_(NCMe)]^+^ where R means Me (**85a**, red line) and Et (**85b**, blue line) [2].

As shown in Scheme 37, the 14-electron structures **85** are involved in the process. Due to their high tendency to fulfill the empty coordination site, these species strongly favor intramolecular contacts. Indeed, it has been experimentally observed that the complexes **S5** bearing R = Et, *n*-Pr and *n*-Bu groups react much faster than other derivatives with R groups without β-hydrogen atoms [61]. This phenomenon has been called the β-hydrogen kinetic effect [2]. It is defined by an incipient β-agostic interaction that can stabilize transient T-shaped intermediates and transition states, and therefore, an increase of the reaction rate would be expected. Further computational studies were performed on complexes [Pt(R)(PMe_3_)_2_(solv)]^+^ **85** describing the isomerization process in different solvents [2]. The β-hydrogen kinetic effect can be detected during the first steps of the isomerization energy profiles (Figure 20) for both the methyl (**85a**, red line) and the ethyl (**85b**, blue line) complexes in acetonitrile. Note that the agostic contact in *cis*-**85b** decreases the overall energy requirement with respect to *cis*-**85a**, but at the same time, it increases the relative energy barrier to reach the Y-shaped transition state **TS85**, i.e., 4.3 kcal mol^−1^ for the ethyl (**TS85b**) and 2.3 kcal mol^−1^ for the methyl (**TS85a**) derivatives. By removal of the agostic interaction in *cis*-**85b** (through a rotation of the C–C bond of the ethyl ligand), this energy stabilization was estimated to be 5.9 kcal mol^−1^. Other solvents such as methanol, dimethylsulfoxide and benzene were also considered. The donor ability strongly affects the dissociation step, although it has a little impact on the strength of the agostic interaction.

**Figure 21:**
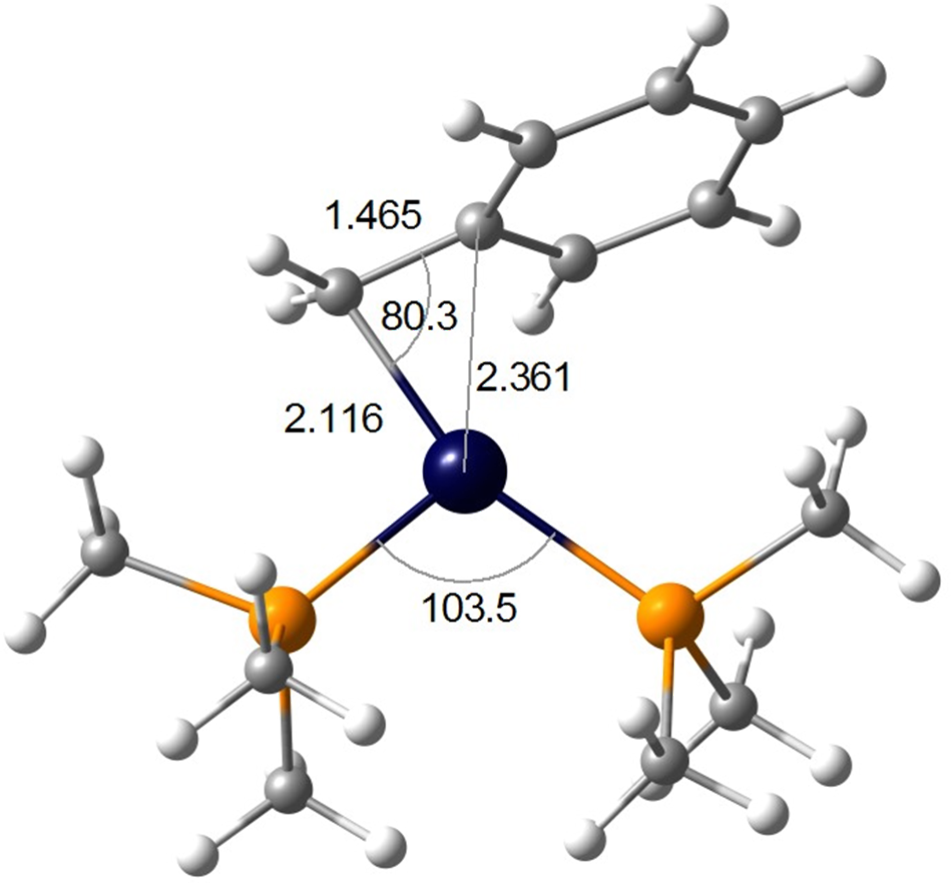
DFT-optimized structure of intermediate **86** [62]. Bond distances in angstrom and angles in degrees.

In the presence of benzyl ligands, a similar *cis*–*trans* isomerization via the unsaturated species [Pt(R)(PEt_3_)_2_]^+^ (Scheme 37) has been claimed [62]. DFT calculations on the model derivative [Pt(Bn)(PMe_3_)_2_]^+^ predict a Pt···η^2^-benzyl coordination mode in **86** involving the ipso-carbon of the benzyl ligand (Figure 21). This stabilization accounts for 7.6 kcal mol^−1^, i.e., 1.7 kcal mol^−1^ stronger than the above-mentioned agostic contact for the same family of compounds (5.9 kcal mol^−1^, [2]). The interaction is favored by electron-donating groups on the phenyl ring and, as a consequence, the reaction rate is enhanced.

### Dissociative ligand substitution

**Scheme 38:**
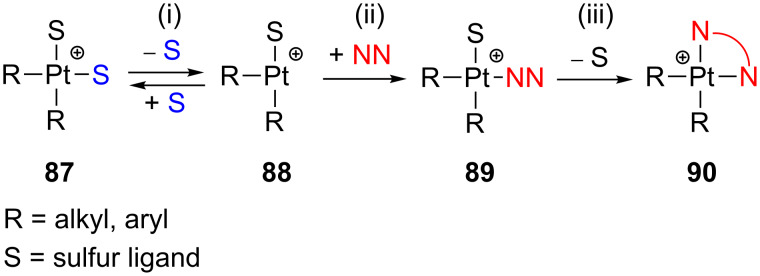
Proposed dissociative ligand-substitution mechanism of *cis*-[Pt(R)_2_S_2_] complexes (**87**) [117].

Experimental evidences obtained in poorly coordinating solvents strongly support that neutral complexes of the type *cis*-[Pt(R)_2_S_2_] (**87**) undergo ligand substitutions through dissociative pathways as depicted in Scheme 38: (i) sulfur ligand dissociation/reassociation via transient 14-electron structures (**88**), (ii) subsequent ligand addition (**89**), and (iii) displacement of the sulfur ligand (**90**) [117,119]. The electron-rich metal, the Pt–S bond weakening due to the *trans*-influence of the R groups, and the stabilization of T-shaped intermediates favor this dissociative pathway. Interestingly, for the complex *cis*-[Pt(Ph)_2_(CO)(SMe_2_)], in which one thioether ligand has been replaced by CO, the operating mechanism becomes associative [145]. Steric and β-hydrogen kinetic effects [2] have been invoked to explain the fast reaction of [Pt(Hbph)_2_(DMSO)_2_] (Hbph = η^1^-biphenyl monoanion) compared to species containing other R groups [117].

**Scheme 39:**
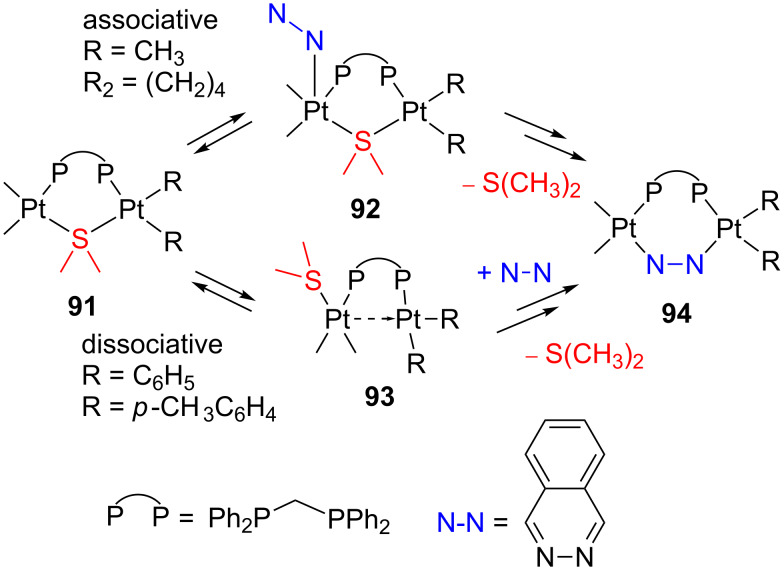
Proposed mechanisms for the ligand substitution of the dinuclear species **91** [146].

Puddephatt and co-workers have demonstrated that, depending on the nature of the R group, dissociative mechanisms can also facilitate the substitution of dimethylsulfide by phthalazine in the dinuclear species **91** (Scheme 39) [146]. When alkyl derivatives are employed, kinetic evidence mainly points to an associative mechanism via **92**, although a minor part of the reaction has been related to a dissociative pathway. The inclusion of phenyl and *p*-tolyl ligands in **91** completely changes the mechanism. A first-order reaction is observed and large negative entropies of activation are collected. The proposed mechanism involves the dissociation of dimethylsulfide generating the putative unsaturated intermediate **93**. This species is supposed to be stabilized by a donor–aceptor Pt–Pt bond, i.e., the electron-rich four-coordinate platinum atom interacts with the electron-deficient three-coordinate counterpart. Consequently, the electron density of the former platinum atom decreases, thereby favoring the subsequent attack of the N–N ligand to yield **94**.

## Conclusion

There are only a very few authenticated three-coordinate Pt(II) complexes. Yet, T-shaped intermediates play an important role in a number of organometallic transformations. Given the electronic and coordinative unsaturated nature of such compounds, they can only be isolated when the vacant coordination site is blocked to avoid intra- and intermolecular interactions. Steric effects are important in preventing the coordination of a fourth external ligand. The use of bulky species, which hamper the entry of ligands into the platinum coordination sphere, is a good strategy toward this goal. The presence of shorter or less flexible alkyl chains in bulky ligands could avoid agostic interactions. Electronic effects, as well as the involvement of strong electron-donating ligands, also have a stabilizing impact. Overall, the number of true T-shaped complexes that are well-characterized is still very low. Nevertheless, a much larger number of Pt(II) complexes can be described as operationally three-coordinate in a kinetic sense. This happens when the fourth position in a square-planar complex is occupied by a very weak ligand, which can be easily displaced. We have named these compounds masked T-shaped complexes. An intramolecular agostic interaction, a weakly coordinated solvent molecule or a counteranion can play such a role. Whether or not the interaction of the platinum atom with the fourth ligand is very weak, the three-coordinate complex should be very close in energy to the square-planar ground state. Consequently, in practice the masked T-shaped complexes can be devised as a resting state of the three-coordinate species.

The accessibility of three-coordinate complexes makes them suitable intermediates in Pt(II) chemistry. In spite of the difficulty in detecting these intermediates, this review gathers a number of recent experimental and theoretical reports, which claim the involvement of T-shaped Pt(II) intermediates in reaction mechanisms. In the near future, the acquired knowledge on the synthetic routes and structural features, as well as the advances in the detection techniques and computational methods, should lead to an improved design of such compounds and to a wider recognition of the role they play in chemical transformations.
